# Genetic analysis of early phenology in lentil identifies distinct loci controlling component traits

**DOI:** 10.1093/jxb/erac107

**Published:** 2022-03-15

**Authors:** Vinodan Rajandran, Raul Ortega, Jacqueline K Vander Schoor, Jakob B Butler, Jules S Freeman, Valerie F G Hecht, Willie Erskine, Ian C Murfet, Kirstin E Bett, James L Weller

**Affiliations:** School of Natural Sciences, University of Tasmania, Private Bag 55, Hobart, TAS 7001, Australia; School of Natural Sciences, University of Tasmania, Private Bag 55, Hobart, TAS 7001, Australia; School of Natural Sciences, University of Tasmania, Private Bag 55, Hobart, TAS 7001, Australia; School of Natural Sciences, University of Tasmania, Private Bag 55, Hobart, TAS 7001, Australia; School of Natural Sciences, University of Tasmania, Private Bag 55, Hobart, TAS 7001, Australia; Forest Genetics and Biotechnology, Scion, Private Bag 3020, Rotorua 3046, New Zealand; School of Natural Sciences, University of Tasmania, Private Bag 55, Hobart, TAS 7001, Australia; School of Agriculture and Environment and Institute of Agriculture, The University of Western Australia, 35 Stirling Highway, Crawley, WA 6009, Australia; School of Natural Sciences, University of Tasmania, Private Bag 55, Hobart, TAS 7001, Australia; Department of Plant Sciences, University of Saskatchewan, Saskatoon, SK, S7N 5A8, Canada; School of Natural Sciences, University of Tasmania, Private Bag 55, Hobart, TAS 7001, Australia; Indian Institute of Science, India

**Keywords:** Adaptation, controlled environment, florigen, flowering time, FT genes, legume, lentil, mapping, photoperiod, QTL analysis

## Abstract

Modern-day domesticated lentil germplasm is generally considered to form three broad adaptation groups: Mediterranean, South Asian, and northern temperate, which correspond to the major global production environments. Reproductive phenology plays a key role in lentil adaptation to this diverse ecogeographic variation. Here, we dissect the characteristic earliness of the *pilosae* ecotype, suited to the typically short cropping season of South Asian environments. We identified two loci, *DTF6a* and *DTF6b*, at which dominant alleles confer early flowering, and we show that *DTF6a* alone is sufficient to confer early flowering under extremely short photoperiods. Genomic synteny confirmed the presence of a conserved cluster of three florigen (*FT*) gene orthologues among potential candidate genes, and expression analysis in near-isogenic material showed that the early allele is associated with a strong derepression of the *FTa1* gene in particular. Sequence analysis revealed a 7.4 kb deletion in the *FTa1*–*FTa2* intergenic region in the *pilosae* parent, and a wide survey of >350 accessions with diverse origin showed that the *dtf6a* allele is predominant in South Asian material. Collectively, these results contribute to understanding the molecular basis of global adaptation in lentil, and further emphasize the importance of this conserved genomic region for adaptation in temperate legumes generally.

## Teaser

A gain-of-function variant at a conserved *FT*locus confers the early phenology in lentil that is critical for adaptation to major production regions in South Asia.

## Introduction

Lentil (*Lens culinaris*) is a grain legume that is widely cultivated around the world and is a staple source of dietary protein and fibre in many countries (Iqbal *et al.*, 2006). Global production is dominated by Canada and India, but substantial production also occurs in Australia, Turkey, and Nepal (FAO, 2020). As in many crop species, appropriate phenology in lentil is a critical determinant of yield and is an important consideration in the development of varieties suited to particular production environments (Hodges, 1990; Kumar *et al.*, 2016).

Lentil was domesticated in the Fertile Crescent from the wild species *L. orientalis*, a vernalization-responsive long-day species with a winter annual habit. This phenology was retained in domesticated lentil, suiting it to an autumn sowing cycle. Its expansion west around the Mediterranean and east into central Asia is likely to have required no major change in phenology. However, its subsequent spread south to the Indian subcontinent and Ethiopia, and north to the Caucasus and beyond, probably depended on significant phenological changes suited to different seasonal climates and cropping patterns.

Some insight into this variation was provided by physiological studies which distinguished independent influences of photoperiod and temperature across diverse lentil accessions and described flowering behaviour with a simple photothermal model (Summerfield *et al.*, 1985; Erskine *et al.*, 1990; Wright *et al.*, 2021). These studies also observed variation in relative responsiveness to photoperiod and temperature, as well as in time to flower under the most inductive conditions, identifying several lines showing minimal acceleration of flowering with increases to temperature, and several others relatively unresponsive to photoperiod (Erskine *et al.*, 1990). A third factor broadly important in the phenology of many temperate annual species, vernalization requirement, is relatively unexplored in lentil, and it is currently not clear how it may relate to photoperiod and temperature responsiveness (Summerfield *et al.*, 1985).

There are currently considered to be three broad adaptation groups of domesticated lentil germplasm; Mediterranean, South Asian, and northern temperate groups, which correspond approximately to the major ecogeographic environments in global production. Adaptation to these environments is associated with differences in phenology that reflect differences in relative sensitivity to photoperiod and temperature (Erskine *et al.*, 1994). For instance, when lentil landraces originating from West Asia are grown in Pakistan and India they flower later than local material, which seems to be generally less sensitive to photoperiod and more sensitive to temperature (Erskine and Saxena, 1993; Erskine *et al.*, 1994). This reduced sensitivity to photoperiod in South Asian landraces is suggested to improve adaptation at lower latitudes by ensuring that flowering occurs at shorter daylength, thus minimizing risk of exposure to late season drought and accommodating the crop efficiently within an intense production system (Erskine *et al.*, 1994).

Such differences in phenology remain a significant barrier to the use of exotic material for germplasm improvement, and a better understanding of their genetic basis and environmental drivers is of high importance for breeding. For example, the narrow genetic base of South Asian germplasm has been substantially broadened by the introduction of elite higher yielding lines that carry an exotic source of photoperiod-insensitive flowering and were thereby pre-adapted to the local environment (Erskine *et al.*, 1998; Sarker *et al.*, 1999). Another example can be seen in the integration of climate data, the photothermal model, and knowledge of flowering time diversity to facilitate selection of genotypes suited to winter sowing in the highlands of central and eastern Turkey (Keatinge *et al*., 1995, 1996).

In the South Asian case, the novel genetics behind the alternative adaptation were shown to derive primarily from a recessive allele at the *Sn* locus (Sarker *et al.*, 1999), which was subsequently identified as the lentil orthologue of the Arabidopsis circadian clock gene *ELF3* (Weller *et al.*, 2012). However, little is known about the genetic basis for the adaptation of the indigenous South Asian material (also referred to as the *pilosae* ecotype). In the present study, we first aimed to characterize the variation in flowering response to photoperiod and vernalization of 16 lentil accessions representing the full range of variation in photoperiod response (Erskine *et al.*, 1990). We then sought to define the genetic basis for the distinct early flowering of a representative South Asian accession.

## Materials and methods

### Plant material and growth conditions

Fifteen accessions of *Lens culinaris* Medik. and one accession of its putative wild progenitor *L. orientalis* (Boiss.) Handel-Mazetti were obtained from the collection at the International Center for Agricultural Research in Dry Areas (ICARDA) (Supplementary Table S1). For each accession, 24 seeds were scarified and sown two per pot in 140 mm pots, in a 1:1 mix of vermiculite and dolerite chips topped with ~3 cm of sterile nursery-grade potting mix with controlled-release fertilizer and granulated sand. Half of the seeds were subjected to a 32 d vernalization treatment at 4 °C, applied to imbibed seed. Unvernalized seeds were sown several days prior to the end of this period in order to synchronize emergence in the two treatment groups. Material was then grown in short (SD; 12 h) or long (LD; 16 h) day conditions, with six plants subjected to each factorial combination of photoperiod and vernalization treatments. All plants received 8 h of natural daylight in a heated glasshouse (mean day temperature 23 °C) before automated daily transfer to night compartments held at 16 °C in which they received a 4 h photoperiod extension with white light from a combination of fluorescent tubes (50 µmol m^–2^ s^–1^) and incandescent globes (5 µmol m^–2^ s^–1^). This extension was followed either by 12 h of darkness (SD) or a further 4 h extension with light from the incandescent globes alone (LD). Plants were lightly watered regularly, and a nutrient solution was applied weekly.

For genetic mapping and association analysis, an F_2_ population (*n*=173) was generated from the cross between the early-flowering Indian landrace ILL 2601 and the late-flowering accession ILL 5588 (cv. Northfield). This population was sown in February 2013 (with growing medium and maintenance as described above) at the University of Tasmania phytotron and evaluated under a SD photoperiod as described above, while parental accessions were in addition evaluated under a LD photoperiod. A total of nine traits related to phenology, plant architecture, and seedling emergence were evaluated during the growing season for use in analysis of quantitative trait loci (QTLs).

Three pairs of F_4_ near-isogenic lines (NILs) segregating for either *DTF6a* (NIL9 and NIL52) or *DTF6b* (NIL85) were developed from progeny of single F_2_ individuals derived from the cross ILL 2601×ILL 5588, by marker-assisted selection of appropriate recombinants in subsequent generations.

### Genotyping, linkage map construction, and QTL analysis

Genotyping was performed by Diversity Array Technology Pty. Ltd (Canberra, Australia) using DArTseq technology (Sansaloni *et al.*, 2011), which generates 64 bp of sequence at each marker by next-generation sequencing. The 173 individuals of the ILL 2601×ILL 5588 F_2_ mapping population were genotyped with a total of 9315 markers. Markers that were identified to be heterozygous for either parent, non-polymorphic, or with >2% missing data were excluded from subsequent analysis. A subset of 2161 polymorphic markers were retained and utilized in the construction of the linkage map.

A genetic linkage map was constructed for the mapping population using JoinMap 4.0 (Van Ooijen, 2006), employing markers without significant segregation distortion and <95% similarity to any other marker. A minimum logarithm of odds (LOD) value of 10.0 was used as the significance threshold to assign markers to groups. The regression algorithm was applied with the Kosambi mapping function and default settings to estimate the order of the polymorphic markers and the distances between markers within each group. After the first iteration of regression mapping, markers within ±1 cM of another marker were manually removed (to reduce marker numbers in high-density regions) and another iteration of regression mapping was employed. Markers with a nearest neighbour fit of >6 cM or a genotype probability <5.0 [–log_10_(P)] were progressively excluded from each linkage group (LG). In LGs with a larger number of excluded markers (>20% of total markers), a secondary attempt at map construction was undertaken including markers with segregation distortion and high similarity. The nomenclature proposed for the LGs is adapted from Sharpe *et al.* (2013). The linkage map was visualized using MapChart 2.3 (Voorrips, 2002). To ascertain the syntenic relationship of the lentil LGs obtained in this study with that of *Medicago truncatula*, the sequences of the DArTseq markers incorporated into the final framework of the ILL 2601×ILL 5588 genetic linkage map were used in a BLAST search against the Medicago reference genome version Mt4.0 (Tang *et al.*, 2014).

QTL analysis on the traits scored in the F_2_ population was undertaken using MapQTL 6 (Van Ooijen, 2009). Permutation tests were run to determine LOD significance thresholds at genome-wide levels (1000 permutations) (Churchill and Doerge, 1994). QTL analyses were first performed using interval mapping. For each putative QTL exceeding the significance threshold in interval mapping, the marker closest to the QTL peak was chosen as a cofactor for multiple-QTL model (MQM) mapping. Cofactors were initially user-determined and then subject to a likelihood analysis based on backward elimination (*P*<0.05) employed by the automatic cofactor selection function to determine their suitability for MQM analysis. The MQM analyses were performed iteratively until no new QTLs were detected and QTL positions were stable (Van Ooijen, 2009). The effect of the peak marker at each QTL on the associated phenotype was also examined.

### Sequencing of the *FTa1–FTa2* cluster

To sequence the genomic region containing *FTa1* and *FTa2*, an amplicon sequencing approach was used: five (four in the case of those accessions carrying the *dtf6a* allele) overlapping fragments were amplified separately using the primers described in Supplementary Table S2. All PCRs were performed in a final volume of 30 μl containing 150 ng of template DNA, 0.4 μM of each primer, and 15 μl of Ranger Mix (Bioline Australia Pty. Ltd). PCR products were purified using ISOLATE II HT 96 Clean-Up Kit (Bioline Australia Pty. Ltd) and quantified using a Standard Sensitivity Large Fragment Analysis kit in a Fragment Analyzer (Advanced Analytical Technologies, Inc). Paired-end libraries were prepared using a Nextera XT Library Preparation Kit (Illumina Inc.) and sequencing was performed using a 300 cycle MiSeq Reagent kit v2 in a MiSeq system (Illumina Inc).

### Gene expression studies

For the expression analysis, two pairs of NILs segregating at the single locus *DTF6a* were grown under an SD (12 h) photoperiod in an automated phytotron at the University of Tasmania. For quantitative reverse transcription–PCR (qRT–PCR), samples of the most recent fully expanded leaves were harvested 3 weeks after emergence. RNA extraction, cDNA synthesis, and gene expression determination were performed as described in Sussmilch *et al.* (2015) using the primers indicated in Supplementary Table S3. The expression level of tested genes was normalized against *Actin* and *Translation initiation Factor* (*TIF*) using the ΔΔCt method.

## Results

### Variation in lentil responsiveness to photoperiod and vernalization

**Fig. 1.**
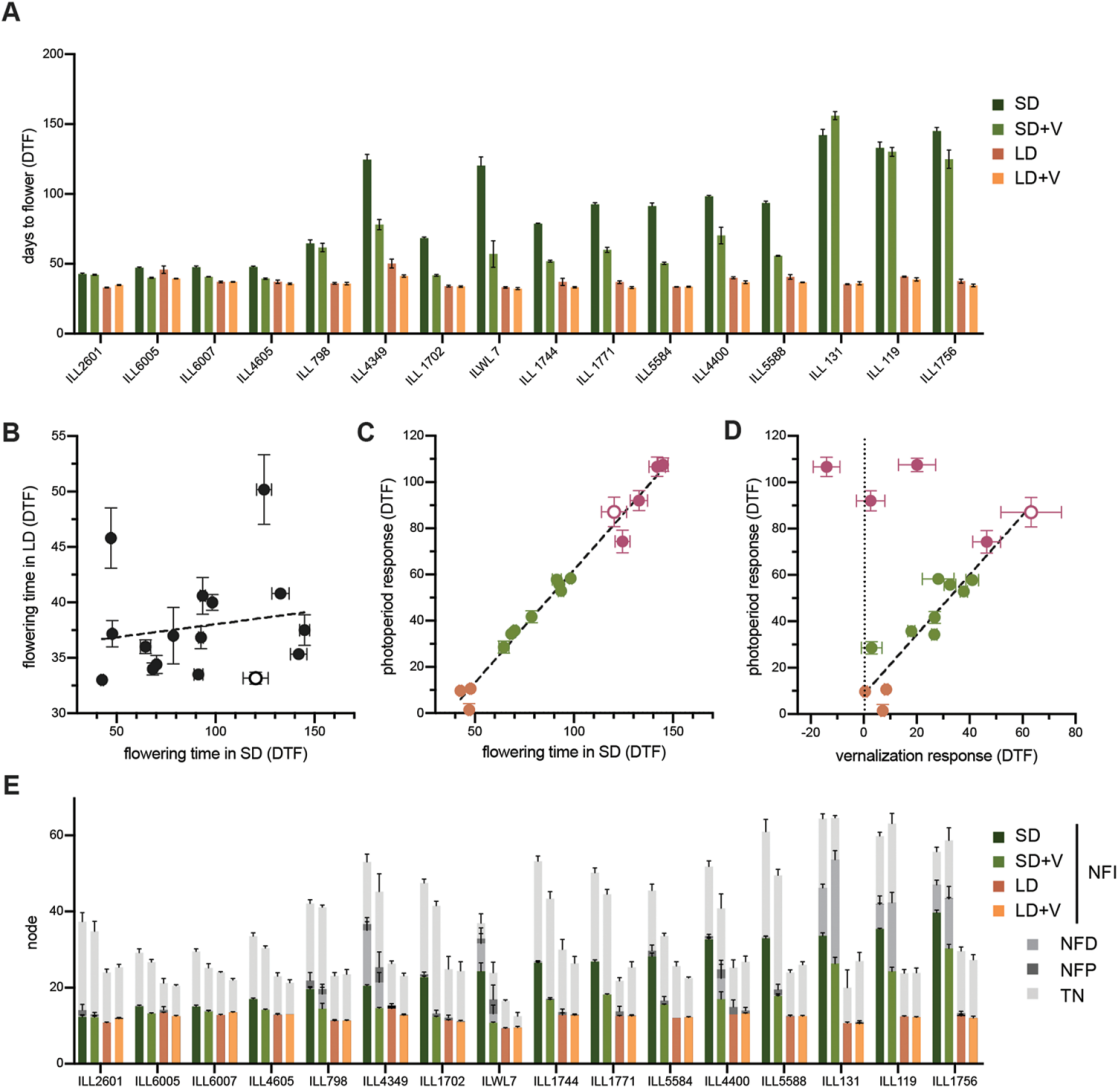
Variation in flowering time and other aspects of reproductive development in response to photoperiod and vernalization. (A) Flowering time of lentil accessions under short- (SD) or long-day (LD) conditions, either with (+V) or without a vernalization treatment. (B) Relationship between flowering time in SDs and LDs. (C) Relationship between photoperiod response (difference in flowering time between SDs and LDs) and flowering time in SDs. Possibly distinct groupings are indicated in orange (early-flowering, low sensitivity), maroon (late-flowering, high sensitivity), and green (intermediate sensitivity). (D) Relationship between photoperiod response and vernalization response for DTF. Colours represent the same groupings as in (C). (E) Phasing of reproductive development. Coloured bars represent the node of flower initiation (NFI). Stacked bars in shades of grey represent the node of first developed flower (NFD), node of first pod (NFP), and the total number of nodes (TN) at maturity (terminal arrest). The wild *L. orientalis* accession ILWL 7 is indicated by open symbols in (A–C). All values represent the mean ±SE for *n*=4–6.

We initially surveyed the variation in flowering time (DTF, the number of days to the opening of the first fully developed flower) and responses to photoperiod and vernalization in a small but diverse collection of lentil accessions representative of major adaptation groups (Supplementary Table S1). Figure 1A shows that these accessions displayed a diverse range of flowering times, ranging from 34 d to 50 d under LDs and from 43 d to 145 d under SDs. Interestingly, flowering times under these two conditions were not correlated (Fig. 1B), suggesting that they are probably subject to independent genetic control. However, flowering time under SDs did show a strong correlation (*r*^2^=0.98) with photoperiod response for DTF (i.e. the difference between DTF in SDs and in LDs), which also varied widely, from 1.4 d in ILL 6005 to >100 d in ILL 131 and ILL 1756 (Fig. 1C). Among the 16 accessions, three (ILL 4605, ILL 6005, and ILL 2601) were distinctively early flowering and showed near insensitivity to photoperiod (Fig. 1C). A second group of five accessions, including the wild *L. orientalis* line ILWL 7, were late flowering with a strong photoperiod response, and the remaining eight accessions showed intermediate responsiveness.

We also examined the response of this panel to a standard vernalization treatment in which imbibed seed were maintained at 4 °C for 4 weeks. The strongest response, a >60 d promotion of flowering, was seen in ILWL 7, and the majority of accessions showed a tight relationship (*r*^2^=0.87) between their degree of responsiveness to photoperiod and vernalization for DTF (Fig. 1D). However, the three accessions that flowered the latest under SDs (ILL 119, ILL 131, and ILL 1756) showed a disproportionately weak vernalization response, potentially identifying them as a distinct response class. Of these, one (ILL 119) had no significant vernalization response and, across all remaining accessions, two others (ILL 4605 and ILL 798) also showed no significant response (Fig. 1D).

In addition to the simple DTF measurement, we also recorded other aspects of reproductive development in these accessions, including the node of initiation of the first floral structure (whether a fully developed flower or an arrested/aborted bud; NFI), the node at which the first flower developed (NFD), the node at which the first pod was produced (NFP), and the total number of nodes at apical arrest (TN). These measurements allowed us to quantify the developmental intervals over which initiated flower buds failed to progress to formation of open flowers and to pods, and the duration of the flowering phase. Flower bud abortion is anecdotally important in several crop legumes (Sita *et al.*, 2017; Maqbool *et al.*, 2010), and in pea has been a characteristic and diagnostic feature of certain allelic combinations for established flowering time loci (Murfet, 1985).

Figure 1E shows that under SD conditions, several lines showed significant abortion of the first formed flower buds and/or fully formed flowers. This tendency was most prominent in the accessions that were late flowering in SDs and showed the strongest photoperiod response for DTF (Fig. 1B), suggesting that these two responses could be a manifestation of a single underlying physiological or genetic system controlling initiation and development.

### Genetic analysis of early flowering in ILL 2601

The reduced photoperiod sensitivity of cv. Precoz (ILL 4605) and derivatives such as ILL 6005 was initially shown to be conferred by recessive alleles at the *Sn* locus (Sarker *et al.*, 1999). In an earlier study, we confirmed this finding in progeny of a cross between ILL 5588 (cv. Northfield) and ILL 6005, and identified a co-segregating mutation in an *ELF3* orthologue as the likely cause of the photoperiod-insensitive phenotype (Weller *et al.*, 2012). Among the accessions examined in Fig. 1, only line ILL 2601 showed a similar dramatic loss of photoperiod sensitivity to Precoz and ILL 6005. However, sequencing of the *ELF3a* gene from ILL 2601 revealed no functionally significant differences relative to ILL 5588 (Supplementary Fig. S1) indicating a genetic basis for earliness independent of *Sn*.

To characterize the genetic control of the earliness in ILL 2601, we crossed it to ILL 5588 and assessed an F_2_ population of 173 individuals under 12 h SD conditions. In addition to the traits described above, we also recorded time to seedling emergence (days to emergence; DTE) given that variation for this trait had been noted in some circumstances previously and could potentially complicate a flowering time assessment based on sowing date alone. Subsequently, we recorded DTF as starting from the date of seedling emergence. We also measured total plant height (PH), number of branches >5 mm in length at 3 weeks post-emergence (number of early branches; EBN), total length of these branches (early branch length; EBL), and the distance between nodes 1 and 9 on the main stem (INL).

**Fig. 2.**
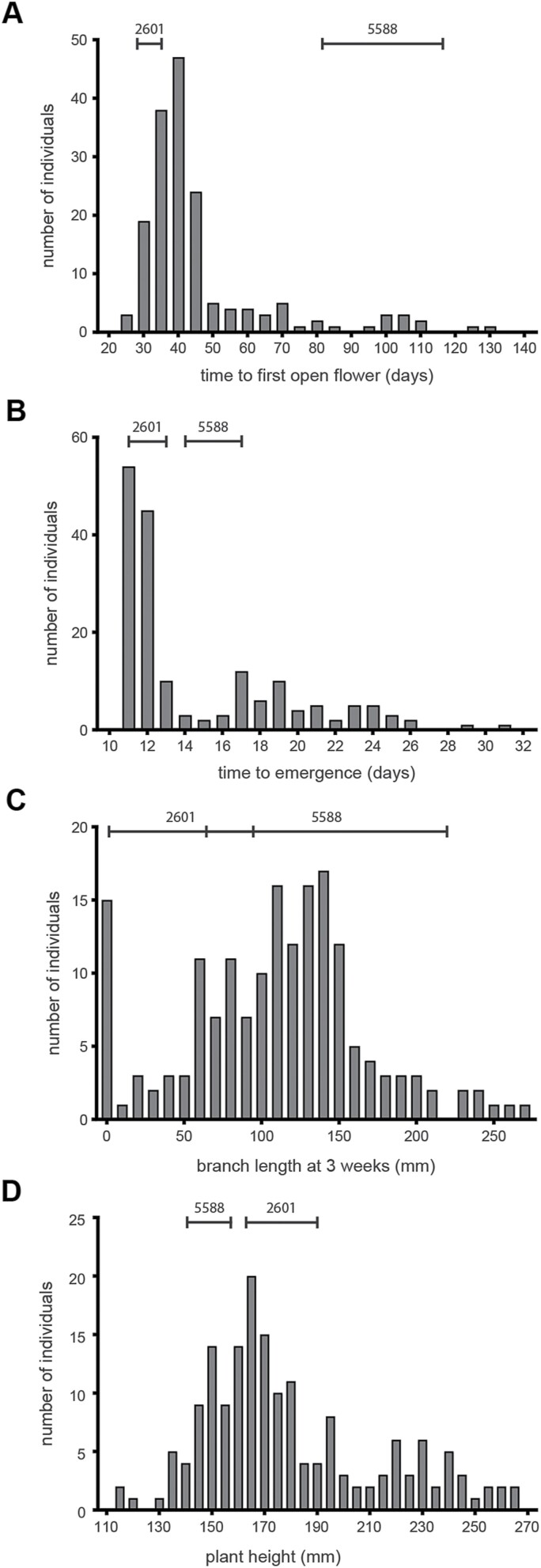
Frequency distributions for phenology and growth habit traits in an F_2_ population. Days to flower (A), days from sowing to seedling emergence (B), total branch length at 3 weeks post-emergence (C), and total plant height (D) were recorded in the F_2_ progeny of a cross between accessions ILL 2601 and ILL 5588. The ranges in parental values are indicated by horizontal bars.

In the F_2_ population, DTF showed an essentially continuous distribution (Fig. 2A), although skewed towards the early phenotype of ILL 2601. This suggests the presence in ILL 2601 of at least one dominant allele conferring early flowering. In addition, the distribution showed a degree of transgressive segregation relative to the parental range in both directions, suggesting the presence of other minor loci potentially involved in the control of DTF. A strong correlation (*R*^2^=0.905) was found between DTF and NFD, but the correlations between DTF and NFI (*R*^2^=0.407) and NFI and NFD (*R*^2^=0.4875) were weaker, reflecting the occurrence of early flower abortion in several individuals (Supplementary Fig. S2).

Analysis of the traits DTE, EBL, and PH in the F_2_ generation showed a continuous distribution (Fig. 2B–D) confirming these as quantitative characters, although there was an indication of bimodality for DTE and PH, implying the involvement of major loci. For both DTE and PH, the range of values in the population was much wider than that in the parental lines, indicating a recombination of parental alleles at multiple loci. For DTE, only late transgressive segregants were recorded, indicating that the allelic combination present in ILL 2601 specified the minimal emergence time, but also included alleles capable of contributing to delayed emergence.

### Linkage mapping and QTL analysis

To further investigate the genetic control of the observed variations, we constructed a genetic linkage map for the ILL 2601×ILL 5588 F_2_ population using a total of 734 DArTseq markers (Supplementary Table S4; Supplementary Fig. S3). The final map has an overall length of 1032 cM, defined by seven LGs that correspond to the seven chromosomes in the lentil genome (2*n*=14). The average distance between adjacent markers was 1.41 cM, with only one interval greater than 10 cM (Supplementary Table S5).

**Fig. 3.**
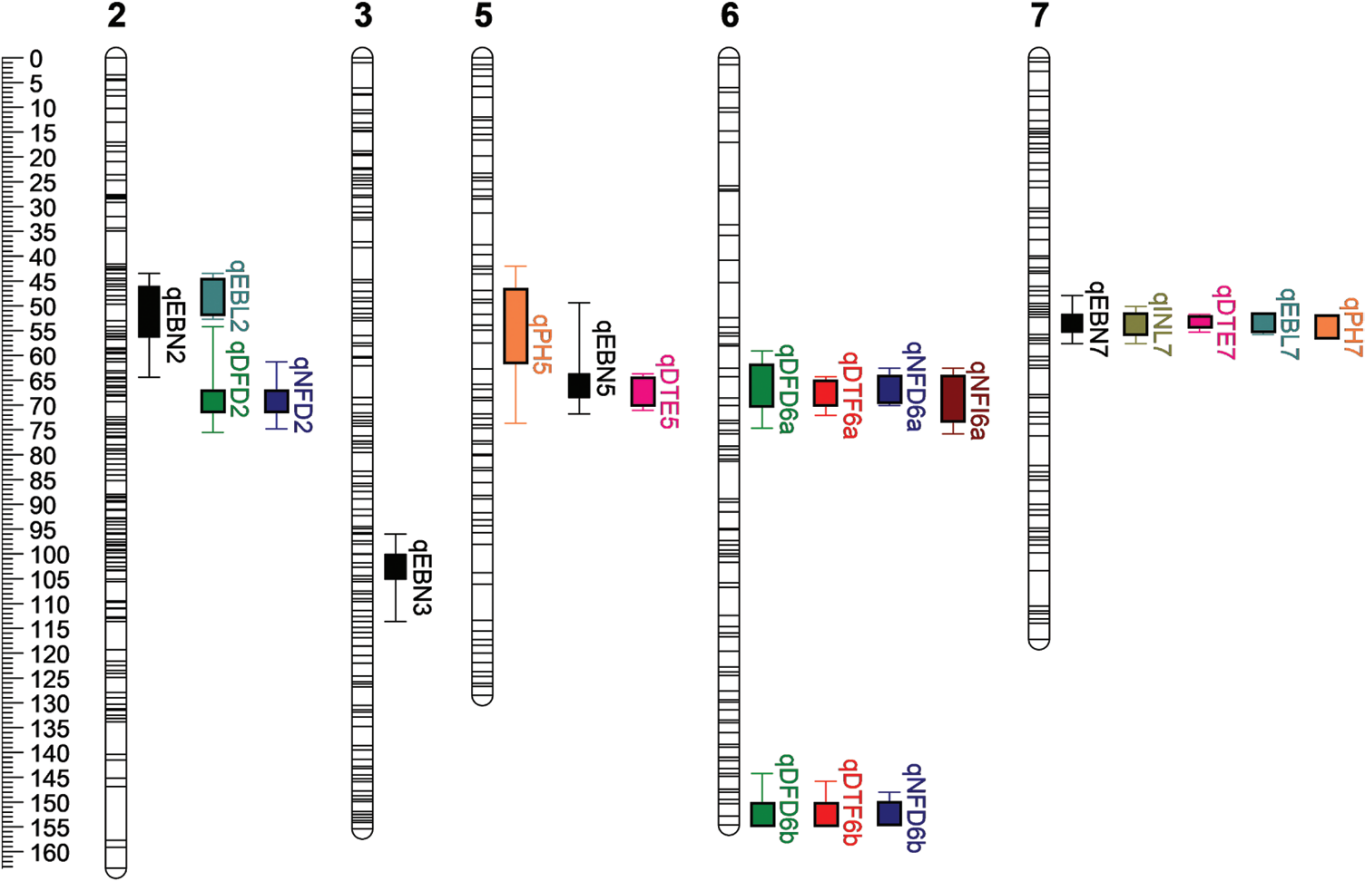
Linkage map showing QTLs detected in the ILL 2601×ILL 5588 population. Scale is cM. QTL nomenclature follows Tables 1 and 2. Box and whiskers represent 1–LOD and 2–LOD intervals, respectively, around each QTL peak.

QTL analysis on flowering traits yielded a total of nine QTLs distributed on LG6 and LG2 (Table 1); of these, seven were detected on LG6. The NFI was associated with only one locus, in a central region of LG6 (LG6A). This region also influenced DTF along with a second distinct region on LG6 (LG6B) (Fig. 3). Together with a third region on LG2, both LG6A and LG6B regions also contributed to control the NFD and the initiation–development interval (DFD). In general, the effects of the QTLs detected on LG6 were stronger than those detected on LG2 for each trait, with estimates of observed variation explained as high as 46% (DTF) or 49% (NFD) when QTLs for these traits in LG6 are combined. In comparison, the LOD and percent phenotypic variance values obtained for the QTLs in LG2 were lower, indicating a contribution of only ~10%.

**Table 1.** Quantitative trait loci for flowering time traits detected in the ILL 2601×ILL 5588 progeny

Trait	QTL	LG	QTL peak position (cM)	LOD	Variation explained (%)	Peak marker	Marker position (cM)
Days to flowering (DTF)	*qDTF6a*​	6	67.3	17.6	33.9	101290509	68.6
	*qDTF6b*	6	152.3	7.1	12.0	3659911	152.8
Node of flower development (NFD)	*qNFD6a*	6	67.3	13.1	23.4	101290509	68.6
	*qNFD6b*	6	152.3	8.7	15.0	3659911	152.8
	*qNFD2*	2	68.1	6.4	10.5	3632005	68.1
Delay to flower development (DFD)	*qDFD6a*	6	67.3	6.3	12.6	101290509	68.6
	*qDFD6b*	6	152.3	7.8	16.1	3659911	152.8
	*qDFD2*	2	68.1	4.2	8.2	3632005	68.1
Node of floral initiation (NFI)	*qNFI6a*	6	67.3	8.0	20.0	101290509	68.6

Three of the four QTLs in the LG6A region (*qDTF6a*, *qNFD6a*, and *qNFI6a*) are the largest contributors to their respective traits, estimated to explain >20% of the observed variation in each case (Table 1). QTLs in the second LG6B region had mostly smaller but still substantial effects, explaining an estimated 12–16% of the observed variation. In both regions, QTLs were observed either to occur at the same position (peak marker) or were co-located within ±1 cM (Fig. 3) and, given the physiologically related nature of the traits concerned, it seems most likely that they represent a single underlying molecular cause in each region. Although this conclusion must be proven empirically, for convenience we will provisionally refer to these two loci as *DTF6a* and *DTF6b*.

Eleven QTLs were detected for traits related to seedling emergence and plant architecture (Table 2); two each for PH, DTE, and EBL, four for the EBN, and one for length between nodes 1 and 9 on the main stem (INL). Of these QTLs, five were located on LG7 in the same interval. In this region, the QTLs *qDTE7*, *qPH7*, and *qINL7* each explained >20% of the observed variation.

**Table 2.** Quantitative trait loci for other traits detected in the ILL 2601×ILL 5588 progeny

Trait	QTL	LG	QTL peak position (cM)	LOD	Variation explained (%)	Peak marker	Marker position (cM)
Days to emergence (DTE)	*qDTE7*	7	53.3	35.4	53.7	3631532	52.3
	*qDTE5*	5	68.5	11.5	12.6	3635263	68.5
Number of early branches (EBN)	*qEBN7*	7	53.3	7.9	14.5	3631532	52.3
	*qEBN5*	5	65.8	5.4	9.6	3634578	65.8
	*qEBN3*	3	101.8	4.9	8.6	3630041	101.8
	*qEBN2*	2	49.7	4.7	8.3	3634748	49.7
Total length of early branches (EBL)	*qEBL2*	2	47.8	6.5	14.3	3630240	46.8
	*qEBL7*	7	52.3	5.2	11.1	3631532	52.3
Plant height (PH)	*qPH7*	7	55.3	14.8	30.6	3630555	55.8
	*qPH5*	5	52.7	4.4	7.9	3634509	53.9
Length between nodes 1 and 9 on the main stem (INL)	*qINL7*	7	53.3	8.4	20.4	3631532	52.3

### Interaction between *DTF6a* and *DTF6b*

To better understand the nature and functional relationship of *DTF6a* and *DTF6b* in control of flowering time, we next examined their individual allelic effects and their interaction in the F_2_ population, as inferred from peak marker genotypes. In view of the strong photoperiod response of the wild *L. orientalis* and of *L. culinaris* accessions from the wider domestication region (Fig. 1; Supplementary Table S1), the alleles from ILL 5588 can reasonably be considered ancestral, and for the purpose of clarity will be referred to as *DTF6a* and *DTF6b*, and the presumed derived alleles from ILL 2601 as *dtf6a* and *dtf6b*.

**Fig. 4.**
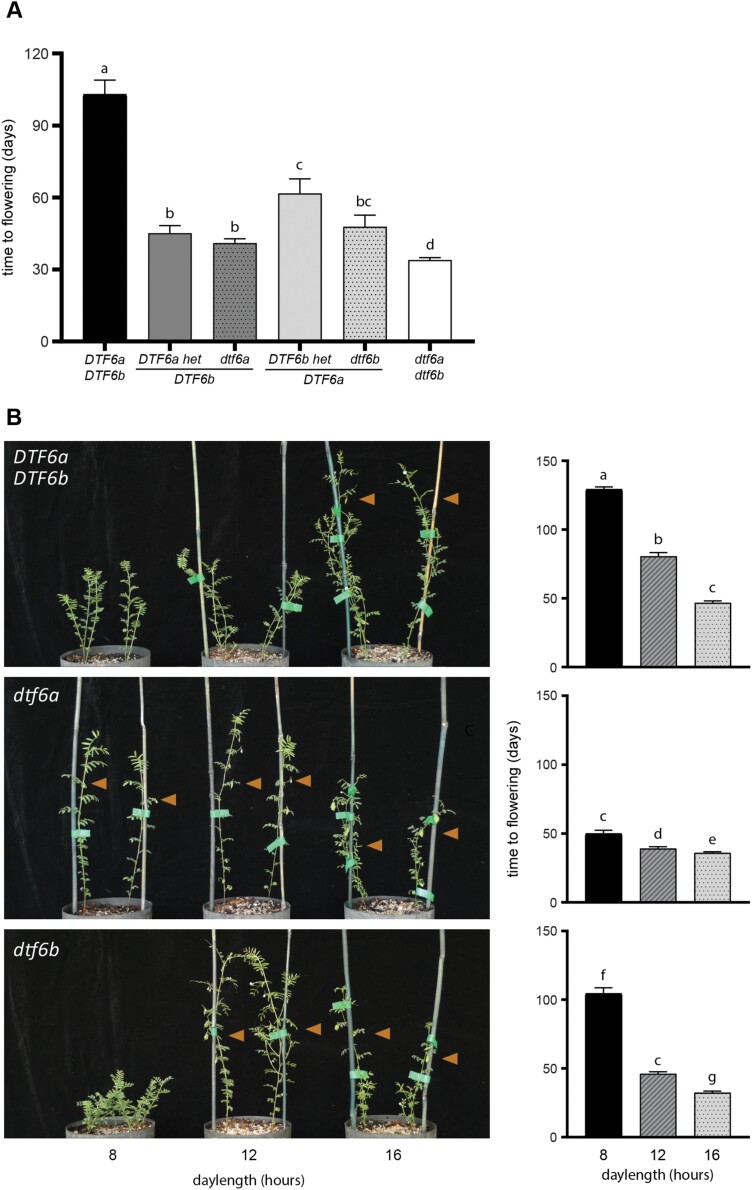
*qDTF6a* and *qDTF6b* peak marker effects under different photoperiods. (A) Interaction between *DTF6a* and *DTF6b* for flowering time in the ILL 2601×ILL 5588 F_2_ population under short-day conditions (12 h). (B) F_4_ NILs differing only at the *DTF6a* (middle panel) or *DTF6b* (bottom panel) locus grown under three different photoperiods: SDs (8 h), SDs (12 h), and LDs (16 h). Plants in the top panel carry ILL 5588 alleles at both loci. Orange triangles indicate the plants that have initiated flowering, with flowering time data shown on the right.

With a *DTF6b* genetic background, F_2_ individuals heterozygous for *DTF6a* did not differ significantly from *dtf6a* homozygous segregants for DTF. Similarly, with a *DTF6a* background, the mean DTF of the *DTF6b dtf6b* heterozygous progeny showed no significant difference compared with *dtf6b* homozygous individuals (Fig. 4A). Taken together, these results indicate a dominant mode of inheritance of the early flowering variants in the SD conditions tested.

The genetic interaction of the two loci was assessed in the F_2_ progeny by comparing the mean DTF of the six genotypic classes. The data in Fig. 4A show that *DTF6a DTF6b* double homozygous progeny flowered significantly later (*P*<0.05) than each of the other genotypic classes. While the individual effects of the *dtf6a* and *dtf6b* alleles were indistinguishable (*P*=0.345), *dtf6a dtf6b* individuals did flower significantly earlier than those with any other allelic combination (*P*<0.05).

For more detailed investigation of the individual effects of *DTF6a* and *DTF6b*, we developed NILs differing at either *DTF6a* or *DTF6b*, and examined them under three different photoperiods: 8, 12, or 16 h (Fig. 4B). Under all three photoperiod conditions, the *dtf6a* and *dtf6b* genotypes conferred significantly earlier flowering than *DTF6a DTF6b*, but their effects were much stronger under the two SD photoperiods (minimum 40 d promotion) than under LDs (10 d earlier), indicating that both loci influenced photoperiod sensitivity. Under 12 h, the effects of the individual loci were similar to those seen in the F_2_ population grown under the same conditions. Both *dtf6a* and *dtf6b* genotypes were significantly earlier flowering than *DTF6a DTF6b* plants (*P*<0.001 for both), although in this case the *dtf6a* line was slightly earlier than *dtf6b* (*P*<0.001). However, under the more extreme SD of 8 h, the d*tf6a* line showed only a minor additional delay in flowering of ~10 d, whereas *DTF6a DTF6b* and *dtf6b* plants showed a more substantial delay of >50 d. This result suggests that of the two ILL 2601 alleles, *dtf6a* is more effective than *dtf6b* for promotion of flowering under SDs, a conclusion also consistent with the greater contribution of *DTF6a* to the variance observed in the F_2_.

### Characterization of candidate genes

Given that *DTF6a* made the strongest contribution to the control of early flowering in ILL 2601, we chose to investigate its identity further, initially by screening the region for potential candidates. To facilitate the selection of candidates within the region surrounding *qDTF6a*, we first established the macrosyntenic relationships between the seven *L. culinaris* LGs defined in the ILL 2601×ILL 5588 map (Fig. 3) and the eight chromosomes of *M. truncatula* (2*n*=16). The genomes were broadly syntenic (Supplementary Fig. S4), but showed the previously documented translocations between the ends of the lentil LGs 1 and 5, and major inversions in regions of lentil LGs 1 and 7 when compared with *M. truncatula* chromosomes 1 and 8 (Sharpe *et al.*, 2013; Gujaria-Verma *et al.*, 2014; Ramsay *et al.*, 2021, Preprint). These findings are consistent with previous observations and are the most comprehensive analysis of synteny between these two species to date.

**Fig. 5.**
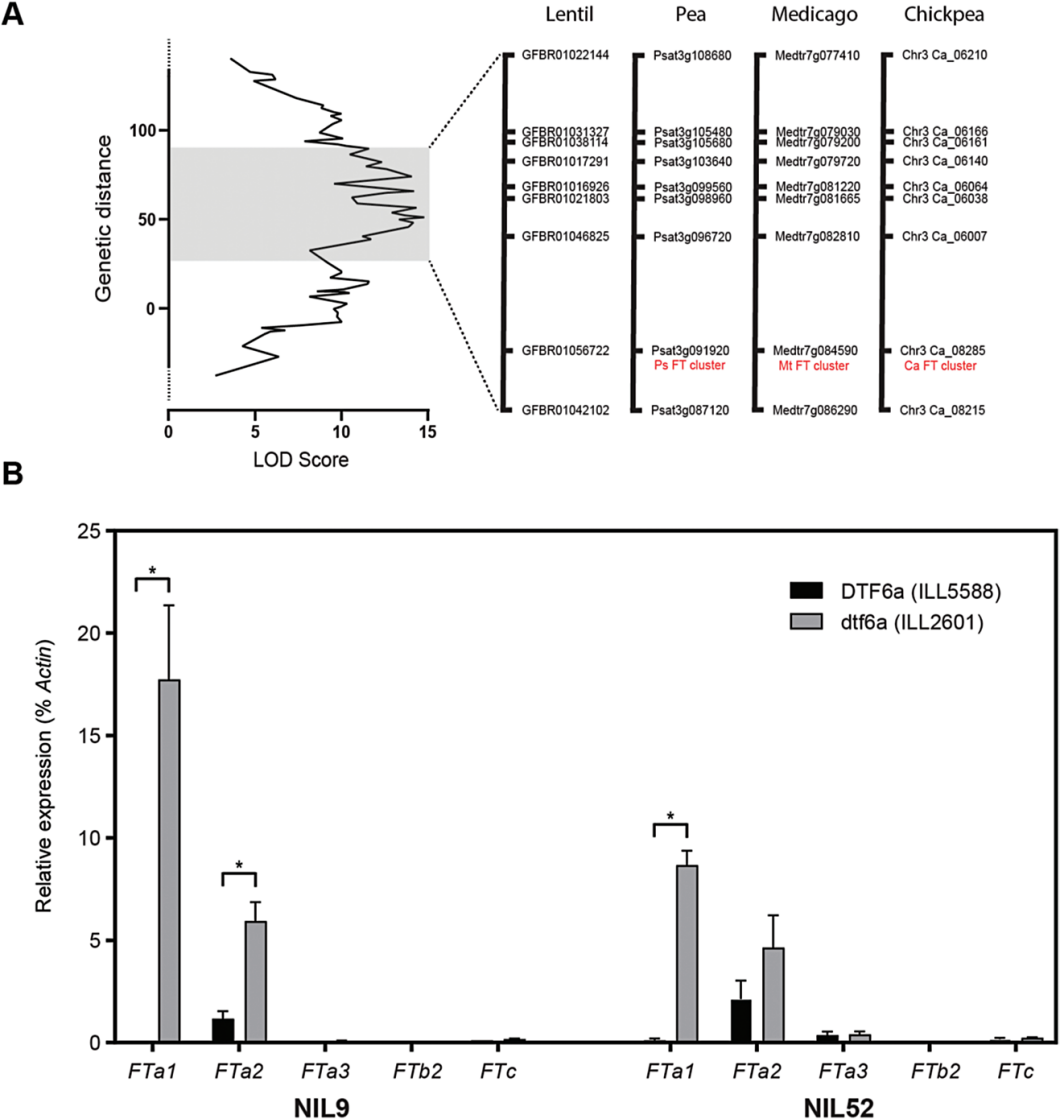
*DTF6a* is located over the conserved *FTa–FTc* cluster and affects its expression. (A) QTL peak for *DTF6a*. Lentil markers in the peak region were blasted against Medicago, chickpea, and pea to determine the matching genes from the syntenic regions. The *FT* cluster of *FTa1*, *FTa2*, and *FTc* is indicated in red. (B) Expression of lentil *FT* orthologues under a short-day (12 h) photoperiod for two pairs of NILs segregating at the single locus, *DTF6a*. Expression levels show the difference between the photoperiod-sensitive ILL 5588 allele (black) and the early-flowering ILL 2601 allele (grey). The most recent fully expanded leaves were harvested 3 weeks after emergence. Values have been normalized to the transcript level of *Actin* and represent the mean ±SE for *n*=3 biological replicates, each consisting of pooled material from two plants.

This comparison indicated that the *DTF6a* region corresponded to an interval of ~4.23 Mbp (627 genes) in the central region of Medicago chromosome 7. This region was also compared with the syntenic regions of chickpea chromosome 3 and pea chromosome 5 (Fig. 5A), revealing the presence of several genes associated in some way with flowering time control and, in particular, three orthologues of the Arabidopsis florigen (*FT*) gene. A conserved cluster of *FT* genes is located in this syntenic genomic region across the two major crop legume clades; and in pea, Medicago, and chickpea the cluster comprises two genes (*FTa1* and *FTa2*) arranged in tandem and a third (*FTc*) located either adjacent or nearby (Supplementary Fig. S5; Supplementary Table S6; Hecht *et al.*, 2011; Laurie *et al.*, 2011; Ortega *et al.*, 2019; Weller *et al.*, 2019). A role for *FT* genes in flowering time control is well documented in many plant species, including legumes (Weller and Ortega, 2015; Lin *et al.*, 2021) and, among the genes in this cluster in the temperate legumes, most evidence points to *FTa1* as being particularly important (Hecht *et al.*, 2011; Laurie *et al.*, 2011; Ortega *et al.*, 2019).

### The *dtf6a* allele is associated with elevated expression of *FTa* genes

Studies of mutants, overexpressors, and natural variation have all indicated that *FTa1* genes promote flowering in proportion to their level of expression (Hecht *et al.*, 2011; Laurie *et al.*, 2011; Ortega *et al.*, 2019). Thus, it is possible that the effect of *dtf6a* might be associated with elevated expression or otherwise increased activity of one or more genes in the cluster in the early parent ILL 2601.

To evaluate this possibility, we compared *FT* gene expression in two independently selected pairs of NILs segregating for *DTF6a*, and evaluated their flowering response to photoperiod. As expected, minor differences were observed in DTF of plants grown under LDs (Supplementary Fig. S6). However, under SDs, *DTF6a* plants were unable to flower before the end of the trial, whereas those bearing the *dtf6a* allele flowered at an average of 69.3 d (NIL9) and 81.2 d (NIL52) after emergence.

The closely related species pea has six *FT* genes (Hecht *et al.*, 2011; Ortega *et al.*, 2019), and an analysis of the recently released lentil genome sequence has confirmed that the same is also true in lentil (Yuan *et al.*, 2021; Supplementary Fig. S5). The results in Fig. 5B and Supplementary Fig. S7 show that in leaf tissue of 3-week-old plants grown under SD conditions, both *FTa1* and *FTa2* genes were expressed at a significantly higher level in NILs carrying the *dtf6a* allele compared with equivalent plants with the *DTF6a* allele. *FTa1* expression was undetectable in *DTF6a* NILs and strongly expressed in the *dtf6a* NILs, whereas *FTa2* was expressed at a low level in *DTF6a* lines and only moderately up-regulated in one of the two NIL comparisons. In contrast, expression of the other four *FT* genes was not reliably detected above background in any of the NILs. These results imply that the early flowering of *dtf6a* lines is likely to result primarily from an increase in *FTa1* expression, and might reflect the impairment of a *cis*-acting mechanism normally acting to maintain repression of *FTa1* expression under non-inductive SD photoperiods.

### Sequence variation in the *FTa1*–*FTa2* cluster

**Fig. 6.**
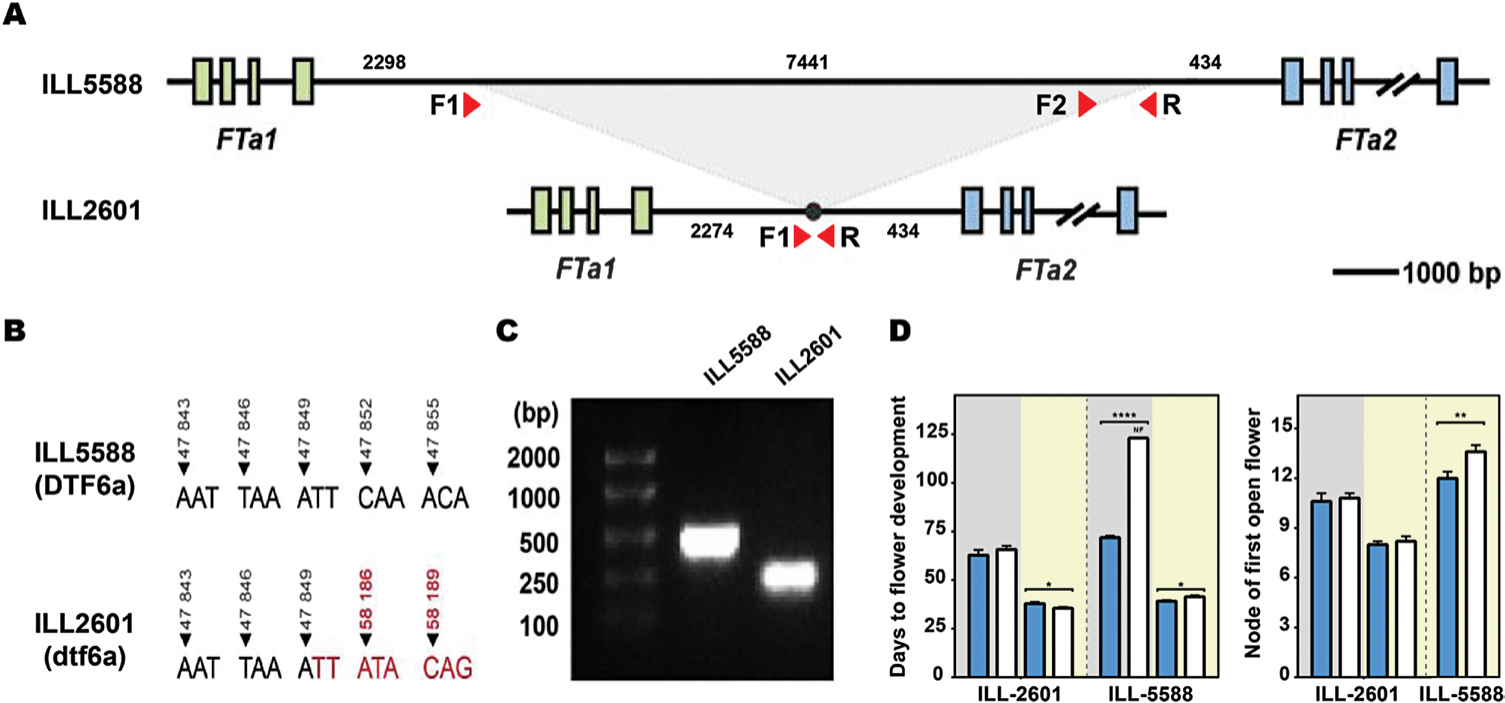
Characterization of the *FTa1–FTa2* cluster in lentil accessions. (A) Schematic diagram of the *FTa1–FTa2* cluster in ILL 5588 and ILL 2601. The green boxes represent the exons of the lentil *FTa1* gene and the blue boxes represent the exons of *FTa2*. (B) The sequence position of the 7441 bp deletion in ILL 2601. (C) PCR of the intergenic region with a 45 s extension time (~1 kb) in ILL 5588 and ILL 2601. Primer positions are annotated in (A). An extended figure including the full gel results is available in Supplementary Fig. S9. (D) Phenotypic response of ILL 5588 and ILL 2601 to photoperiod and vernalization treatment. Blue and white bars represent vernalized and unvernalized plants, respectively. Background colour indicates photoperiod, with SDs (8 h) in grey and LDs (16 h) in yellow. NF indicates plants unable to flower at the end of the scoring period (123 d). Consequently, their flowering node is not present in the corresponding graph.

To identify sequence variation that might be associated with misregulation of the *FTa1* and *FTa2* genes, we sequenced the *FTa1–FTa2* cluster in the parental lines ILL 2601 and ILL 5588 using an amplicon sequencing approach, from ~4.5 kb upstream of *FTa1* to ~1 kb downstream of *FTa2*. The *FTa1* gene is relatively compact, whereas the *FTa2* gene extends to almost 22 kb, with a large third intron >20 kb in length (21 045 bp in ILL 2601, 20 723 bp in ILL 5588). We found no sequence differences in the coding sequence of either gene among the two accessions, but identified a total of 136 single nucleotide polymorphisms (SNPs) and 25 insertions/deletions (indels) distinguishing ILL 5588 and ILL 2601 in non-coding regions (Supplementary Fig. S8; Supplementary Tables S7–S9. By far the most substantial of these was a large (7441 bp) deletion that eliminated most of the *FTa1–FTa2* intergenic region in ILL 2601 (Fig. 6A–C). Another significant polymorphism, due to its potential position within the *FTa1* promoter, is a 245 bp indel found 3712 bp upstream of the *FTa1* start codon (Supplementary Fig. S8; Supplementary Table S7). Other minor variants were found scattered across the cluster, but they were especially abundant within the third intron of *FTa2*, which accumulated 83% of all SNPs and 56% of all indels detected between the two sequences. This contrasts with the high conservation observed for the *FTa1* gene, where no indels and only three SNPs were found within the entire gene (introns included). This contrasting degree of sequence conservation between the two *FTa* genes further supports the idea of *FTa1* having a more relevant role than *FTa2* in lentil.

In Medicago, transposon insertions in the 3ʹ region of *FTa1* are reported to confer an early flowering phenotype that is photoperiod sensitive but not responsive to vernalization (Jaudal *et al.*, 2013). To clarify whether ILL 5588 and ILL 2601 might differ in their response to vernalization, we grew vernalized and unvernalized plants from ILL 5588 and ILL 2601 in either LDs or SDs, using a more extreme (8 h) SD treatment than shown in Fig. 1 (Fig. 6D; Supplementary Table S10). In the late-flowering ILL 5588, vernalization caused earlier flowering under both photoperiods. Under SDs, unvernalized plants remained vegetative after 123 d, whereas vernalized plants flowered at an average of 71.8 d. Under LDs, plants flowered earlier and showed a small (2.3 d, 1.3 nodes) but significant (*P*<0.05) promotion of flowering by vernalization. In contrast, we found no significant reduction in ILL 2601 flowering time due to vernalization in either of the two photoperiods tested. Moreover, under LDs, vernalized plants flowered slightly later than unvernalized plants (2.4 d, *P*<0.05). However, differences in flowering time and flowering node between the two photoperiod treatments were apparent.

The physiological similarity between ILL 2601 and the Medicago mutants described above strengthens the idea that the *FTa1*–*FTa2* intergenic deletion might be the causal polymorphism for the observed *FTa1* up-regulation. In view of its potential significance, we designed a PCR-based specific marker for this polymorphism (Supplementary Table S11; Supplementary Fig. S9), and examined its prevalence in a lentil collection of 48 accessions available at the University of Tasmania and across a broader diversity panel of 324 accessions (Wright *et al.*, 2021), both selected to cover a wide range of geographic origins. Results from the screening were similar in both collections and show that, globally, the deletion is common within lentil germplasm, and 23–37.5% of all accessions carry the deletion (Supplementary Figs S10, S11). However, based on the geographic origin of the accessions, its distribution was uneven: in South Asian material, it was found to be the main allelic variant, present in >75% of accessions, but it was less frequent in accessions of Middle-Eastern origin and rare in germplasm from Mediterranean and temperate environments (Supplementary Figs S10, S11). Independently of whether the deletion or any other polymorphism is responsible for the *dtf6a* effect, the high prevalence of the ILL 2601 allele in South Asian accessions suggests that the *DTF6a* locus might substantially contribute to the earliness characteristic of this material.

## Discussion

Identification of variation for target traits and an understanding of the underlying genetics is important for the development of lentil varieties locally adapted to different agro-economical environments. Despite its global importance as a crop, lentil is chronically understudied in this respect. We characterized natural variation for photoperiod and vernalization response in a small collection of lentils representative of the major adaptation groups, and found wide variation in the flowering response to these cues (Fig. 1), in agreement with earlier studies proposing differential photothermal sensitivity as a mechanism for latitudinal adaptation (Erskine *et al.*, 1989, 1990, 1994). The Indian landrace ILL 2601 was one of the earliest to flower in our survey and has an early phenology typical of the *pilosae* ecotype, which is adapted to the environments of the Indian subcontinent and is characterized by a reduced photoperiod sensitivity and increased responsiveness to temperature (Summerfield *et al.*, 1985; Erskine *et al.*, 1990, 1994). Our QTL analysis, performed on a cross between this line and the photoperiod-sensitive accession ILL 5588 (cv. Northfield), detected two loci on lentil chromosome 6 controlling flowering time (*DTF6a* and *DTF6b*). Both loci show a co-dominant- to dominant-early inheritance pattern (Figs 3, 4A), and are clearly distinct from the previously described *Sn/ELF3a* locus on chromosome 3. Although both *DTF6a* and *DTF6b* contribute in an additive manner to the phenotype of ILL 2601, analysis of segregants in the F_2_ progeny and NILs revealed that the individual effect of the ILL 2601 allele at *DTF6a* is notably stronger than that of the *DTF6b* allele under an 8 h photoperiod (Fig. 4B).

The role of *FT* genes as flowering promoters is widely conserved throughout the plant kingdom (Wickland and Hanzawa, 2015), and this seems to be true also for legumes (Weller and Ortega, 2015; Lin *et al.*, 2021). In several temperate legumes, flowering time loci show a conserved syntenic position near a cluster of *FT* orthologues and, in chickpea and lupin, are also associated with an increased expression of one or more of the underlying genes (Weller and Ortega, 2015; Nelson *et al.*, 2017; Ortega *et al.*, 2019). The confidence interval for the lentil *qDTF6a* locus detected in this study also spans the orthologous *FT* cluster (Fig. 5A) and, as in the other species, there is strong evidence that the allelic difference influences expression of one or more of these genes. In two independent NIL pairs differing at the *DTF6a* locus, *FTa1* showed strong differential expression, with expression undetectable in the late-flowering *DTF6a* genotypes but evident in the early-flowering *dtf6a* (ILL 2601) genotypes. *FTa2* was expressed in *DTF6a* lines and showed only weakly differential expression, while *FTc* expression was negligible in all lines. In addition, none of the other three *FT* genes in other genomic locations was differentially expressed (Fig. 5B).

Functional evidence from pea and Medicago implicates *FTa1* as the most important of the three genes in the cluster. In both species, deleterious polymorphisms within the coding region of *FTa1* result in late flowering, while overexpression in Medicago confers early flowering and reduced sensitivity to both photoperiod and vernalization (Hecht *et al*.. 2011; Laurie *et al*.. 2011). Although we cannot definitively exclude a contribution of *FTa2* to the observed effect of the lentil *DTF6a* locus, the expression change was much weaker and seen only in one NIL pair. In addition, relative to *FTa1* genes, the pea and Medicago *FTa2* genes have minimal capacity to complement an Arabidopsis *ft* mutant (Laurie *et al.*, 2011; Hecht *et al.*, 2011) and, in chickpea, lines with a complete deletion of *FTa2* did not display a significant difference in flowering behaviour (Ortega *et al.*, 2019).

The fact that the early *DTF6a* allele from ILL 2601 shows dominant inheritance and confers increased *FTa1* gene expression is consistent with a scenario in which the normal repression of *FTa1* under non-inductive (i.e. SD and/or unvernalized) conditions has been compromised. In other species, early-flowering variants co-locating with *FT* genes have been variously attributed to alterations in the promoter or other regulatory regions, tandem duplication or increase in copy number, or gain-of-function missense mutations (e.g. Beales *et al.*, 2007; Nitcher *et al.*, 2013). In narrow-leafed lupin (*Lupinus angustifolius*), dominant alleles at the *Ku* locus confer early, vernalization-insensitive flowering, and increased expression of the single underlying *FTc* clade gene, and feature a deletion within its promoter that presumably harbours important repressive elements (Nelson *et al.*, 2017; Taylor *et al.*, 2019). In addition, in Medicago, retroelement insertions in the third intron or in the 3ʹ-untranslated region (UTR) of *FTa1* confer a similar dominantly inherited early-flowering phenotype, and allow expression of *FTa1* to occur in the absence of vernalization (Jaudal *et al.*, 2013). While this could possibly reflect a direct activation by the insertion, it might also represent interference with a repressive mechanism. Among many sequence differences across the *FTa1–FTa2* region (Supplementary Fig. S8), the most prominent was a 7.4 kb deletion in the *FTa1*–*FTa2* intergenic region (Fig. 6) which might plausibly harbour repressive elements acting on *FTa1.* The incidence of this polymorphism in a diversity panel capturing most of the genetic and geographic diversity within cultivated lentil germplasm (Wright *et al.*, 2021) suggests that the *dtf6a* allele is largely restricted to, and strongly enriched in, South Asian germplasm. Although this might partly be attributable to a founder effect, it also seems likely to reflect positive selection, and implies that *DTF6a* may contribute to earliness in the *pilosae* ecotype more generally.

In a recent study parallel to this one, interspecific genetic analysis of flowering time differences between *L. culinaris* and its putative wild ancestor *L. orientalis* also revealed a QTL co-locating with *qDTF6a*, and identified *FTa1* as the only differentially regulated candidate gene within the QTL interval (Yuan *et al.*, 2021). Interestingly, in this case, *FTa1* expression was higher, and flowering was earlier, in the wild parent relative to the domesticated parent cv. Lupa. This implies the existence of another derived variant distinct from that in ILL 2601, potentially involving loss rather than gain of function. Together these results suggest that regulatory and structural changes that alter *FTa1* expression are an important component of natural variation for flowering time in lentil.

### Genetic control of flowering time component phases

The period between sowing and emergence, known as the pre-emergent phase (Roberts *et al.*, 1986), has typically been subsumed within flowering time measurements based on sowing date. However, our genetic analysis identified two loci that influence this interval, that are located in genomic regions independent of those controlling the time from emergence to flowering. Broadly speaking, the pre-emergent phase itself also has several physiological components, incorporating physical and physiological dormancy and early shoot growth. This study excludes any contribution from physical dormancy since all seed coats were scarified. Interestingly, the tight co-location of a QTL for DTE with those for internode length and plant height on chromosome 7 suggests that these traits might all reflect the action of a gene involved in growth rate of the main stem, which would lead to a faster emergence of the shoot, longer internodes, and thus a taller plant.

We also observed differences in the period between flower initiation and flower opening. In general, under LD conditions, the first flower to initiate is also the first to develop and open, but, under SDs, flower buds, once formed, may then fail to develop for a number of nodes (Fig. 1E). This phenomenon is observed in a number of other temperate legumes, including pea and chickpea (Murfet, 1985; Roberts *et al.*, 1985), and exposes a disjunction between the genetic programme specifying the developmental transition to flowering, and the more general physiological orientation of the plant towards reproduction. Interestingly, we were able to associate this tendency specifically with the *DTF6a* region (Fig. 3). Thus, while both *DTF6a* and *DTF6b* govern the node and time of first open flower under SDs, the ILL 2601 allele at *DTF6a* also has an additional effect promoting the early formation of flower buds. These observations are potentially significant because the arrest of floral buds may be one consequence of exposure to environmental stresses in pulse crops, such as cool temperatures in chickpea (Nayyar *et al*., 2005*a*, 2005*b*; Fang *et al.*, 2010). An improved knowledge of its genetic control and its relationship to phenology more generally may help in understanding and managing it.

## Supplementary data

The following supplementary data are available at *JXB* online.

Table S1. List of accessions used in Fig. 1.

Table S2. Details of the primers used for amplicon sequencing of the *FTa1–FTa2* gene cluster in ILL 2601 and ILL 5588.

Table S3. List of primers used in this study for gene expression analysis.

Table S4. Sequence associated with DArT markers in the ILL 2601×ILL 5588 F_2_ genetic linkage map.

Table S5. Statistics of the genetic linkage map displayed in Supplementary Fig. S3.

Table S6. Gene accession numbers of the 48 PEBP amino acid sequences from five plant species used to build the phylogenetic tree displayed in Supplementary Fig. S5.

Table S7. Summary of all the polymorphisms found in the comparison between the *FTa1*–*FTa2* sequences from lentil accessions ILL 5588 and ILL 2601.

Table S8. Description of all the SNPs found in the 38 926 bp length alignment of the *FTa1–FTa2* sequences from lentil accessions ILL 5588 and ILL 2601. 

Table S9. Description of all indels found in the 38 926 bp length alignment between the *FTa1*–*FTa2* sequences from lentil accessions ILL 5588 and ILL 2601.

Table S10. Mean and standard deviation values obtained for days from emergence to first flower and node of the first open flower in lentil lines ILL 2601 and ILL 5588 grown in four different conditions.

Table S11. Primers used in the design of the PCR-based marker of the 7441 bp deletion in the *FTa1*–*FTa2* intergenic region.

Fig. S1. Multiple sequence alignment of the ELF3 proteins in lentil accessions ILL 6005, ILL 5588, and ILL 2601.

Fig. S2. Correlation of traits measured in the ILL 2601×ILL 5588 F_2_ mapping population.

Fig. S3. ILL 2601×ILL 5588 F_2_ genetic linkage map.

Fig. S4. Synteny between the lentil genetic linkage map and the *M. truncatula* genome (Mt4.0).

Fig. S5. Phylogenetic tree of the phosphatidylethanolamine-binding protein gene family in five legume species.

Fig. S6. Flowering phenotype of two pair of NILs segregating for *DTF6a*, evaluated under LD and SD conditions.

Fig. S7. Expression of lentil *FT* orthologes under short day photoperiod for two pair of NILs segregating at the locus *DTF6a*. Expression values normalized to the transcript level of *Translation Initiation Factor*.

Fig. S8. Graphical overview of the alignment between the *FTa1*–*FTa2* sequences from lentil accessions ILL 5588 and ILL 2601.

Fig. S9. Visualization, in a 2% agarose gel, of the PCR products obtained with primers described in Supplementary Table S11 tested in different lentil lines.

Fig. S10. Incidence of the 7441 bp deletion in 48 lentil lines (University of Tasmania lentil collection) with diverse geographical origin.

Fig. S11. Incidence of the 7441 bp deletion in a panel of 324 lentil lines with diverse geographical origin.

## Supplementary Material

erac107_suppl_supplementary_tables_S1-S3_S5-S11_figures_S1-S10

erac107_suppl_supplementary_table_S4

## Data Availability

Raw data that support the findings of this study are available from the corresponding author, upon reasonable request.
