# Supplementary material for: Genetic analysis of early phenology in lentil identifies distinct loci controlling component traits
Source: J Exp Bot. 2022 Mar 15;73(12):3963–77. doi: 10.1093/jxb/erac107 (PMC9238442; doi:10.1093/jxb/erac107)
Supplement: erac107_suppl_supplementary_tables_S1-S3_S5-S11_figures_S1-S10 [file erac107_suppl_supplementary_tables_s1-s3_s5-s11_figures_s1-s10.pdf]

Supplementary Table S1. List of all accessions used in Figure 1.

Accession	Type[a]	Other name(s)	Origin	Latitude[b]	Comments
ILWL 7	Wild	-	Turkey	38°N	
ILL 119	LR	PI 170478	Turkey	37°N	
ILL 131	LR	PI 174251	Turkey	38°N	
ILL 798	LR	F75	Egypt	30°N	
ILL 1702	LR	EL32	Ethiopia	11°N	
ILL 1744	LR	EL 79	Ethiopia	10°N	
ILL 1756	LR	72L	Afghanistan	35°N	
ILL 1771	LR	326L	Afghanistan	35°N	
ILL 2601	LR	LWS 16	India	23°N	
ILL 4349	CV	Laird	Russia	50°N	
ILL4400	LR	Syrian local large	Syra	36°N	
ILL 4605	CV	Precoz	Argentina	27°S	
ILL 5584		78526004	Jordan	32°N	Selection from LR
ILL 5588	CV	Talya 2	Jordan	32°N	Released in Lebanon as Talya 2
ILL 6005					Selection from cross of Laird x Precoz
ILL 6007					Selection from cross of Laird x Precoz

[a] LR = Landrace; CV = Cultivar; Wild = *Lens orientalis*
[b] approximate latitude of origin

Supplementary Table S2. Details of the primers used for amplicon sequencing of the *FTa1-FTa2* gene cluster in ILL 2601 and ILL 5588

		Primer name	Primer Sequence
**Fragment 1**		LcFTa1-15F	TTCGTCGAGTGCATTTTCAA
		LcFTa1-2R	TGCATATAACTAGTGCTTGCTTG
**Fragment 2**	**Deletion lines**	LcFTa1-2F	ACCTCAGGGATCCATCGTTT
		LcFTa2-7R	AGTCCCGGTAGTTTCTGGAAT
	**Normal lines**		
	Fragment 2a	LcFTa1-2F	ACCTCAGGGATCCATCGTTT
		LcFTa1-6R	GATCGATCTGGAATTGGATAAC
	Fragment 2b	LcFTa2-8F	CTCACCCCACCTCACAACTT
		LcFTa2-7R	AGTCCCGGTAGTTTCTGGAAT
**Fragment 3**		LcFTclust-3F	TCTTGTTGTTGGGCGTGTAA
		LcFTa2-8R	ACGGGAAACCCATCATGTTT
**Fragment 4**		LcFTclust-4F	CAAACGTCCATAGCCCTGAT
		LcFTa2-12R	TGCATACACATTGAAGCAAGC

Supplementary Table S3. List of primers used in this study for gene expression analysis.

Target gene	Primer name	Sequence	Reference
FTa1	LcFTa1-8F	CCGATATTCCAGCAACTACTGA	
	LcFTa1-7R	AACACGAACACGAAACGATG	
FTa2	LcFTa2-F2	ACCCCATGATCTACTACACCC	
	LcFTa2-4R	CCATCACAATTCAAAGCAATG	
FTa3	LcFTa3-F1	AGTTCCAGGAATATCAGTCACC	
	LcFTa3-R1	CCAAGGGTTAAGGTTGGTGG	
FTb2	LcFTb-3F	GGTGAACCCTGATGCACCTA	
	LcFTb-1R	GAACGTTGTCCCAGTAGTCG	
FTc	LcFTc-F3	GGTGCATCTGCGTCCACC	
	LcFTc-R3	ACAATTGGTTAATCGTCCAAGGG	
Actin	LcActin-Fw	CCAAATCATGTTTGAGGCTTTTAA	Sen Gupta et al., 2017
	LcActin-Rv	GTGAAAGAACGGCCTGAATAGC	
TIF	LcTIF-Fw	CCGTTGCGCCTATCATTCA	Saha et al., 2012
	LcTIF-Rv	ACGTCTTTCCGGTTCCAGATT	

1. Saha GC, Vandemark GJ. 2012. Evaluation of expression stability of candidate references genes among green and yellow pea cultivars (Pisum sativum L.) subjected to abiotic and biotic stress. American Journal of Plant Sciences 3, 235.

2. Sen Gupta D, McPhee K, Kumar S. 2017. Development of molecular markers for iron metabolism related genes in lentil and their expression analysis under excess iron stress. Frontiers in Plant Science 8, 579.

Supplementary Table S4. Sequence associated with DArT markers in the ILL 2601 x ILL 5588 F_2 genetic linkage map.

Supplementary Table S4 is supplied as an independent excel file.

Supplementary Table S5. Statistics of the genetic linkage map displayed in Supplementary Figure S3.

Linkage group	Markers	Length (cM)	Density (markers/cM)
1	115	158.40	1.38
2	136	163.35	1.20
3	118	155.43	1.32
4	151	154.81	1.03
5	67	128.51	1.92
6	75	154.63	2.06
7	72	117.16	1.63
TOTAL	734	1032.28	1.41

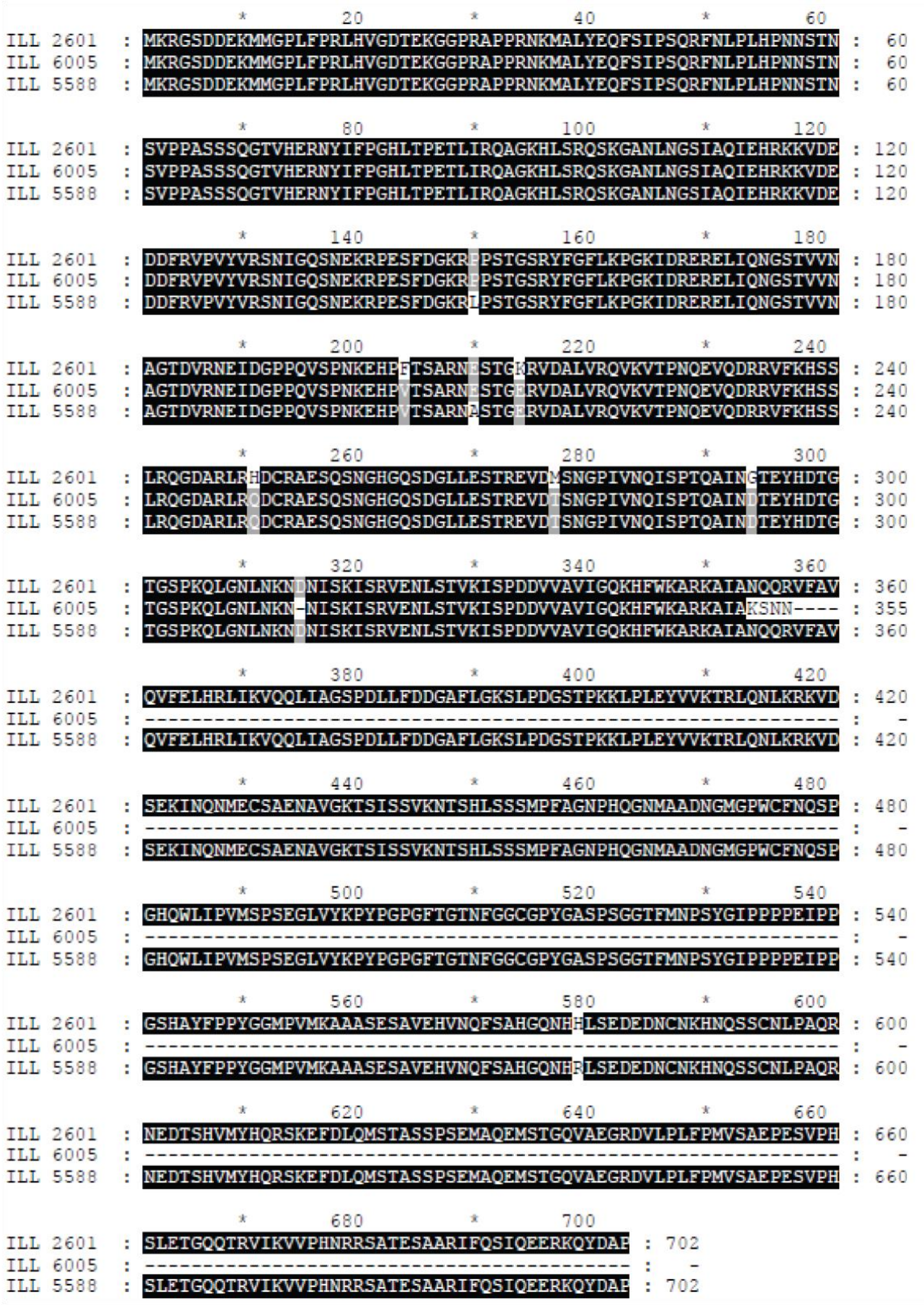

Supplementary Figure S1. Multiple sequence alignment of the ELF3 proteins in lentil accessions ILL 6005, ILL 5588 and ILL 2601. Shades indicate minimum conservation level: Black = 80%, grey = 60%.

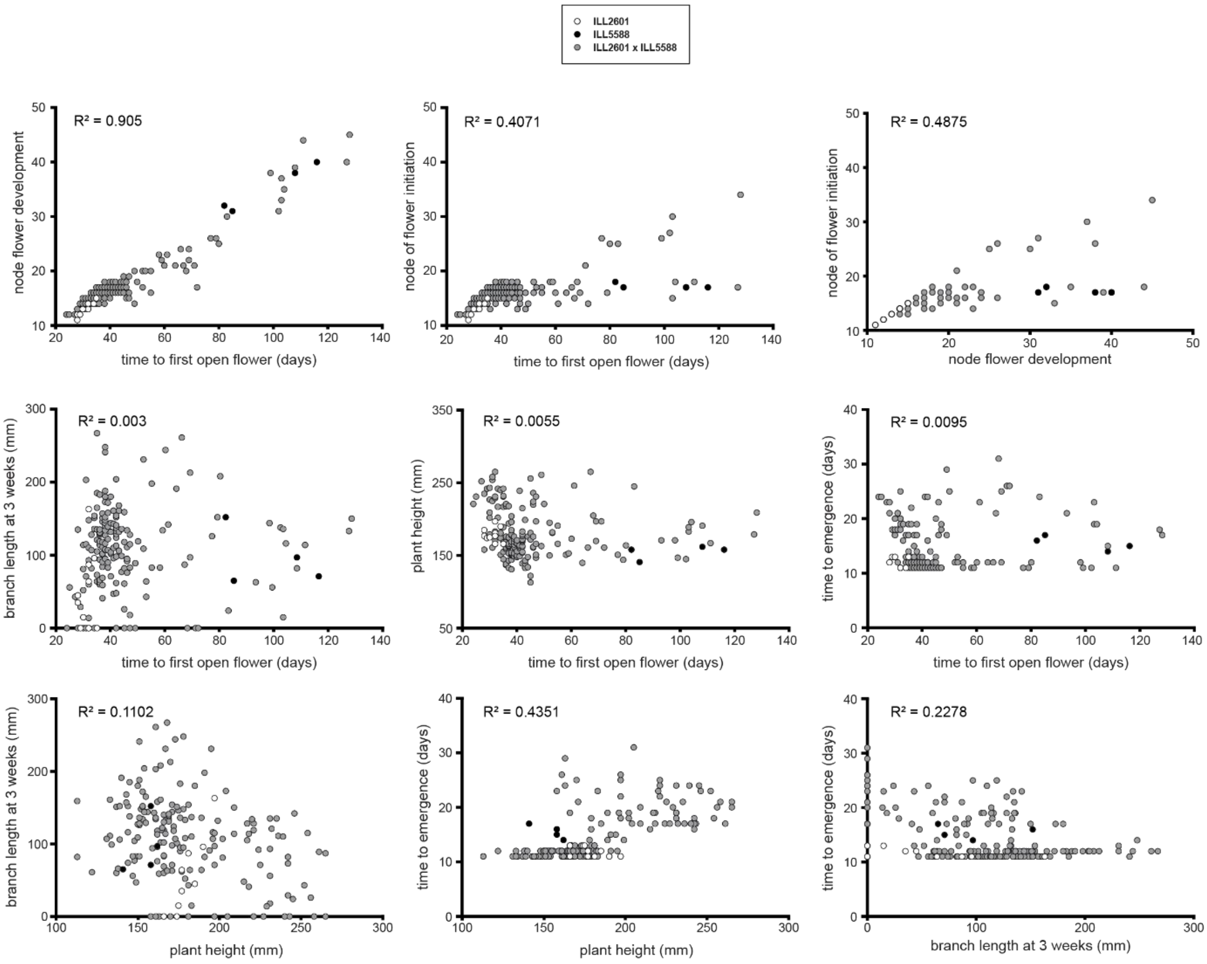

Supplementary Figure S2. Correlation of traits measured in the ILL 2601 x ILL 5588 F_2 mapping population. The reduced correlation of floral initiation with flower development and flowering time is indicative of flower abortion in some lines.

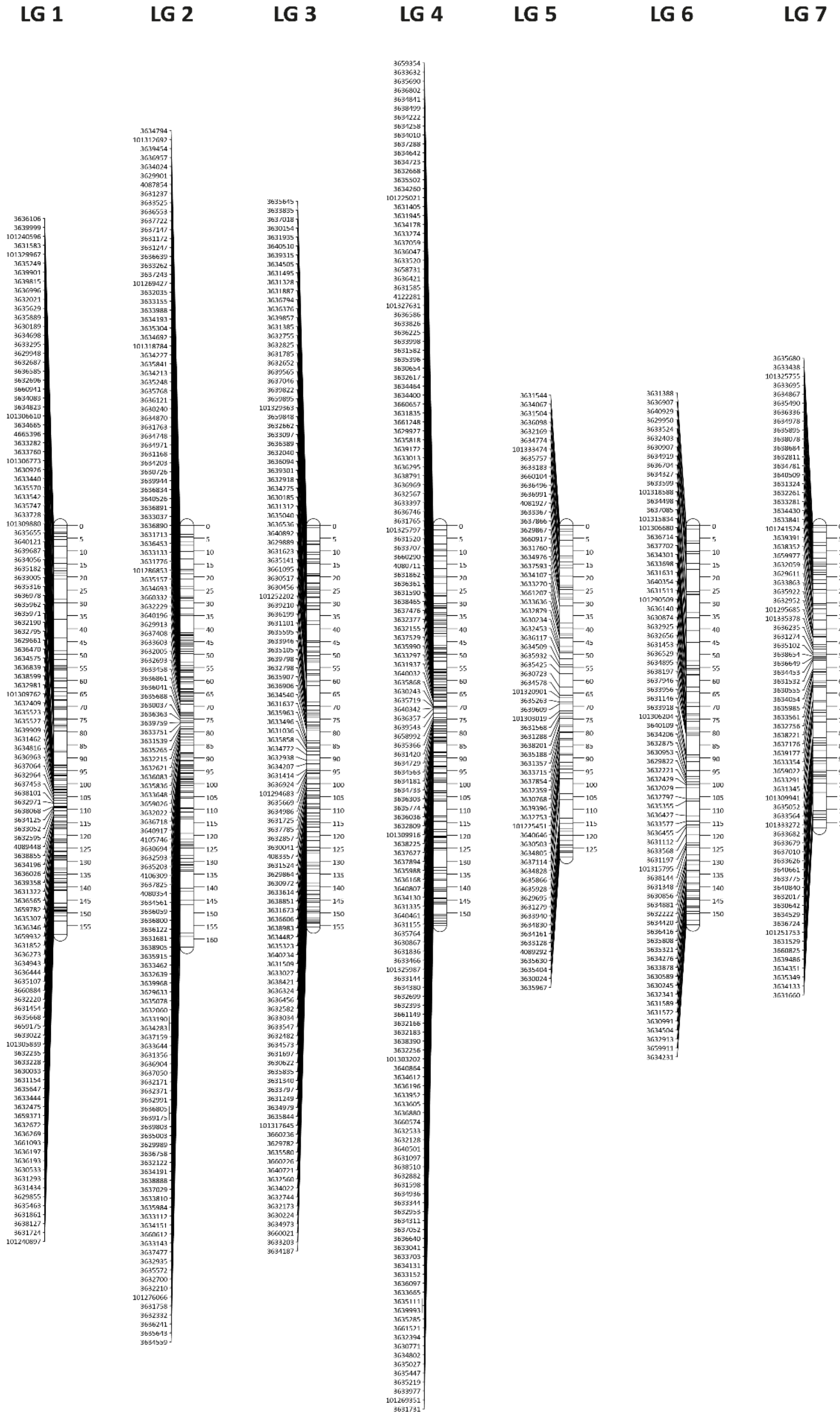

Supplementary Figure S3. ILL 2601 x ILL 5588 F2 genetic linkage map

Genetic linkage map consists of seven linkage groups corresponding to the seven chromosomes of the *Lens* genus. The nomenclature proposed is adapted from Sharpe et al. (2013).

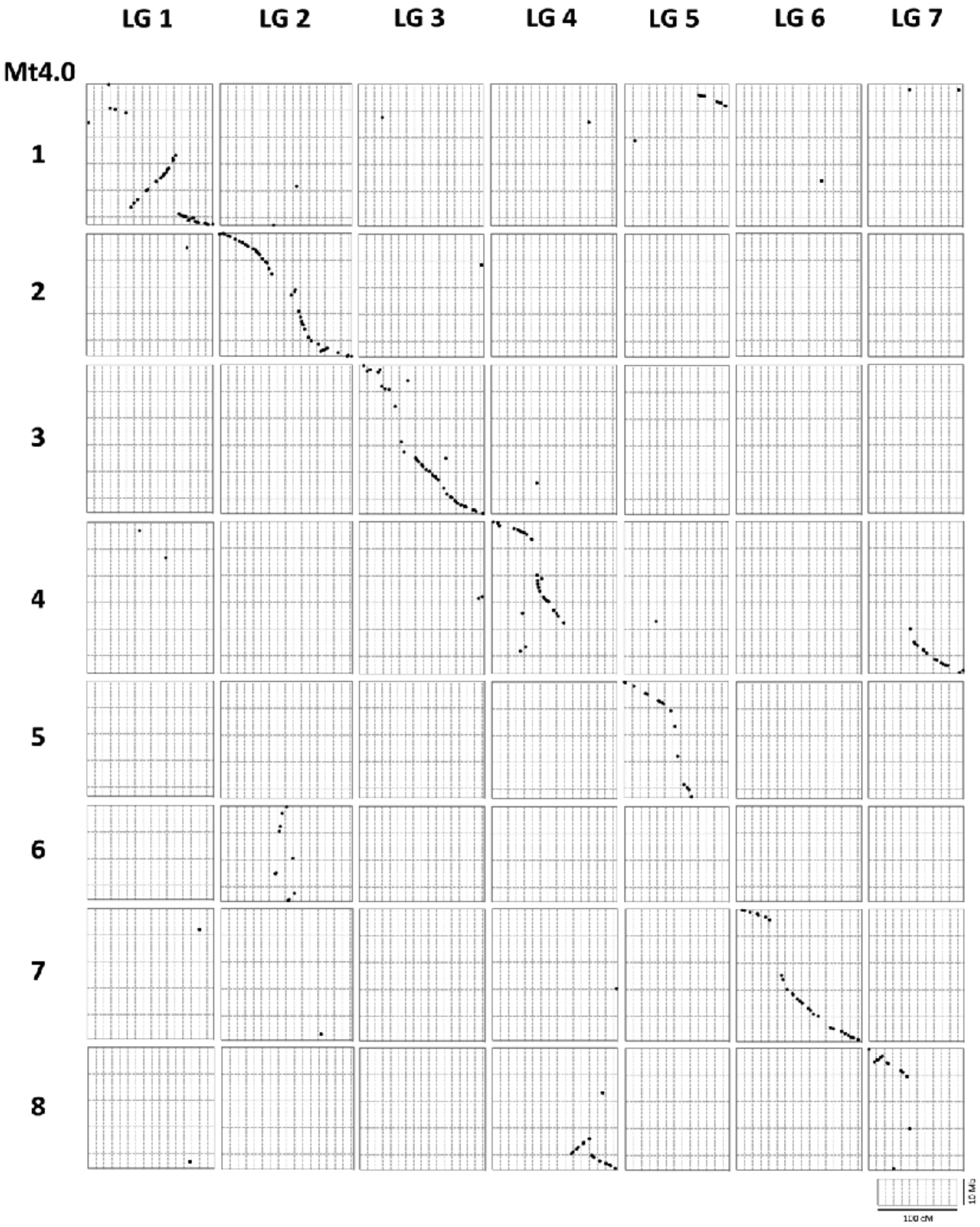

Supplementary Figure S4. Synteny between lentil genetic linkage map and *M. truncatula* genome (Mt4.0)

Each Y-axis interval represents 10 Mb in the Mt4.0 assembly and each X-axis interval represents 10 cM in the linkage map. 256 markers with sequences demonstrating significant similarities (*e*-value < 0.001), and 33 markers (*e*-value < 0.1) and 38 markers (*e*-value < 0.01) with sequences demonstrating low similarities are visualised in the dot plot. The dot plot was manually graphed using Prism 6.

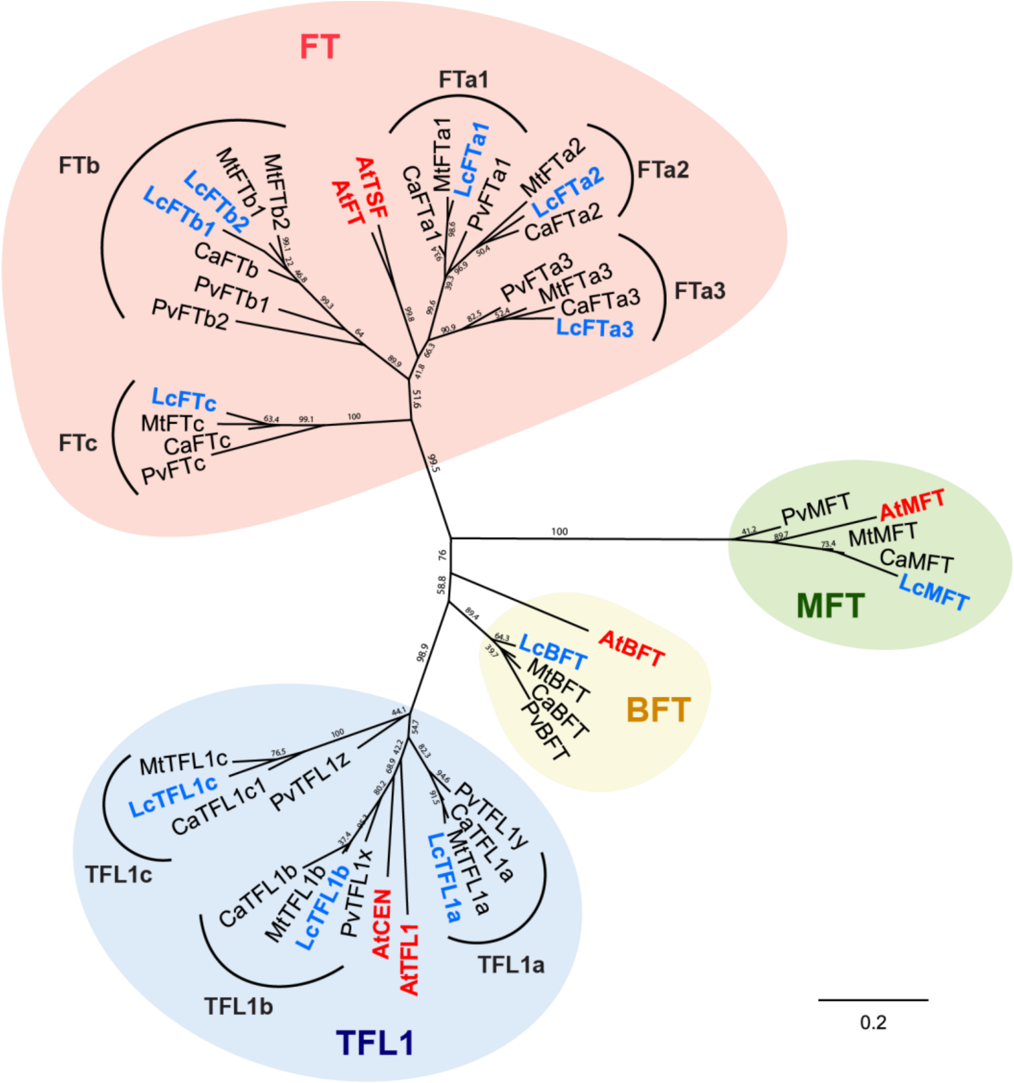

Supplementary Figure S5. Phylogenetic tree of the phosphatidyl ethanolamine-binding protein (PEBP) gene family in five legume species (Lc=*Lens culinaris*, in blue, Mt = *Medicago truncatula*, Ca = *Cicer arietinum*, Pv = *Phaseolus vulgaris*) and *Arabidopsis thaliana* (At, in red). Protein sequences from gene accessions in supplementary table S6 were aligned in Geneious Prime 2020.2.2 (http://www.geneious.com) using MAFFT. Phylogenetic tree was inferred using PhyML .3.20180621 (Guindon et al. 2010) , Blosum62 method and a bootstraping of 1000 replications.

Supplementary Table S6. Gene accession numbers of the 48 PEBP amino acid sequences from five plant species used to build the phylogenetic tree displayed in Supplementary Figure S5.

Arabidopsis	
**Gene**	**Accession**
Arabidopsis thaliana	
AtBFT	AT5G62040
AtCEN	AT2G27550
AtFT	AT1G65480
AtMFT	AT1G18100
AtTFL1	AT5G03840
AtTSF	AT4G20370

Legumes			
**Gene**	**Accession**	**Gene**	**Accession**
Phaseolus vulgaris		***Cicer arietinum***	
PvBFT	Phvul.004G119700	CaBFT	LOC101507903
PvFTa1	Phvul.001G097300	CaFTa1	LOC101497376
PvFTa3	Phvul.004G074700	CaFTa2	LOC101496618
PvFTb1	Phvul.008G003800	CaFTa3	LOC101515383
PvFTb2	Phvul.008G003700	CaFTb	LOC101505276
PvFTc	Phvul.001G097200	CaFTc	LOC101508200
PvMFT	Phvul.002G327700	CaMFT	LOC101504081
PvTFL1x	Phvul.005G124600	CaTFL1a	LOC101506075
PvTFL1y	Phvul.001G189200	CaTFL1b	LOC101508699
PvTFL1z	Phvul.007G229300	CaTFL1c1	LOC101495644
Lens culinaris		***Medicago truncatula***	
LcBFT	Lcu.2RBY.2g038980	MtBFT	Medtr0020s0120
LcFTa1	Lcu.2RBY.6g043850	MtFTa1	Medtr7g084970
LcFTa2	Lcu.2RBY.6g043870	MtFTa2	Medtr7g085020
LcFTa3	Lcu.2RBY.2g046590	MtFTa3	Medtr6g033040
LcFTb1	Lcu.2RBY.6g000730	MtFTb1	Medtr7g006630
LcFTb2	Lcu.2RBY.6g000760	MtFTb2	Medtr7g006690
LcFTc	Lcu.2RBY.6g043940	MtFTc	Medtr7g085040
LcMFT	Lcu.2RBY.4g081710	MtMFT	Medtr8g106840
LcTFL1a	Lcu.2RBY.6g060590	MtTFL1a	Medtr7g104460
LcTFL1b	Lcu.2RBY.2g084140	MtTFL1b	Medtr2g086270
LcTFL1c	Lcu.2RBY.1g033180	MtTFL1c	Medtr1g060190

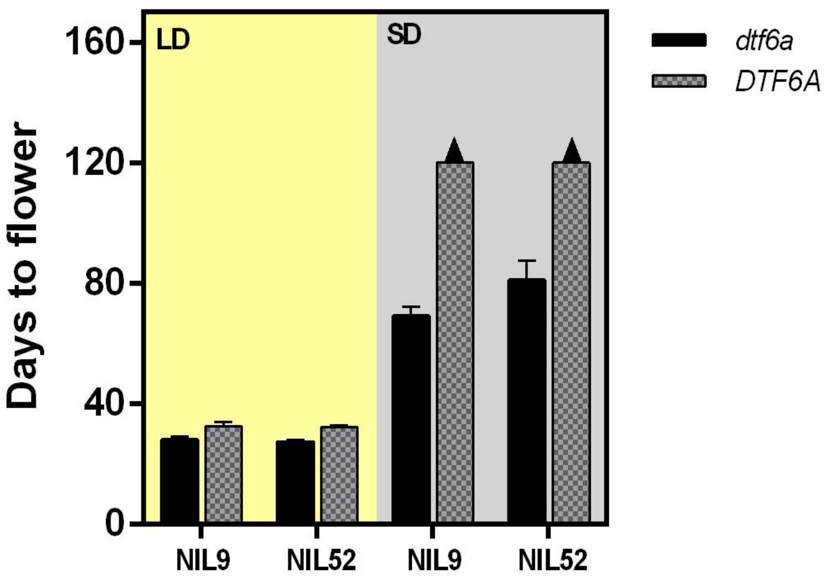

Supplementary Figure S6. Flowering phenotype of two pair of NILs segregating for *DTF6a*, evaluated under LD (16 hours photoperiod, yellow background) and SD (8 hours photoperiod, grey background) conditions. Arrows over the columns indicate that plants were unable to flower after 120 days.

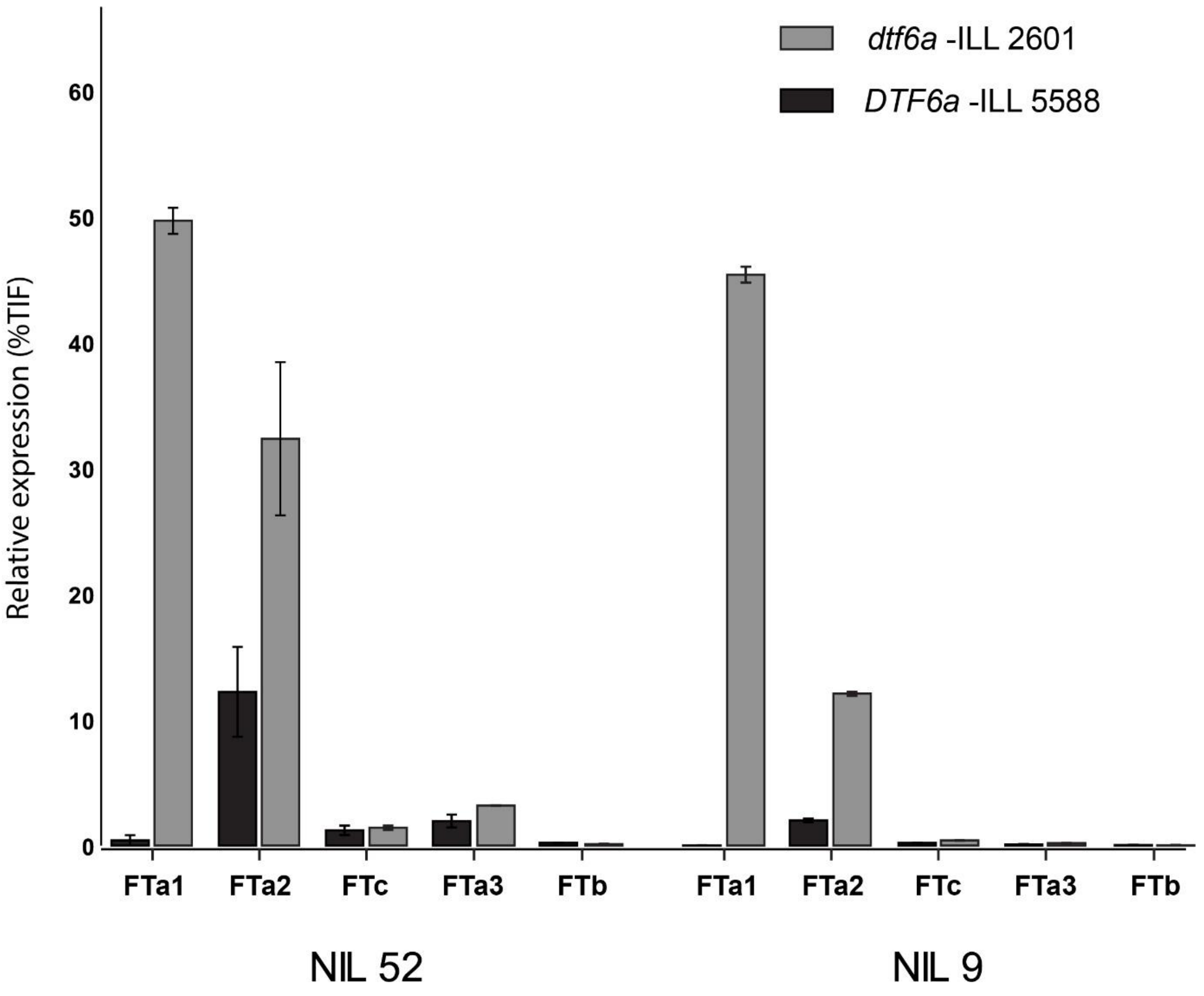

Supplementary Figure S7. Expression of lentil *FT* orthologues under short day (12hr) photoperiod for two pairs of NILs segregating at the single locus, *DTF6a*. Expression levels show the difference between the photoperiod-sensitive ILL 5588 allele (black) and the early-flowering ILL 2601 allele (grey). The most recent fully expanded leaves were harvested three weeks after emergence. Values have been normalised to the transcript level of *Translation Initiation Factor* and represent mean ± SE for n=3 biological replicates, each consisting of pooled material from two plants.

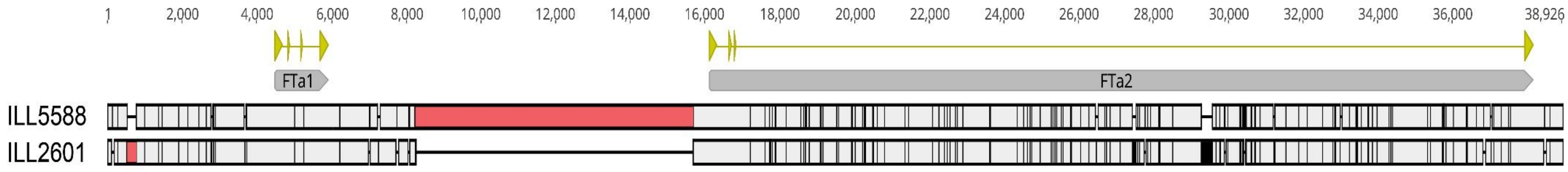

Supplementary Figure S8. Graphical overview of the alignment between the *FTa1-FTa2* sequences from lentil accessions ILL 5588 and ILL 2601. Grey boxes over the alignment indicate the extent of *FTa1* and *FTa2* genes. Yellow triangles represent exons and yellow lines introns. Sequence similarities are shown in grey and polymorphisms in black. Relevant deletions are highlighted in red.

Supplementary table S7. Summary of all the polymorphisms found in the comparison between the *FTa1-FTa2* sequences from lentil accessions ILL 5588 and ILL 2601.

	FTa1 5' UTR	FTa1 Gene	Intergenic	FTa2 Gene	FTa2 3' UTR	Total
Position (alignment)	1-4491	4492-5925	5926-16117	16118-38173	38174-38926	
Size (alignment)	4491	1434	10192	22056	753	38926
Size (ILL 5588)	4230	1434	10173	21652	753	
Size (ILL 2601)	4489	1434	2708	21974	747	31352
SNPs	14	3	5	113	1	136
Indels	5	0	5	14	1	25
Total	19	3	10	127	2	161

Supplementary Table S8. Description of all the SNPs found in the 38926 bp-length alignment of the *FTa1-FTa2* sequences from lentil accessions ILL 5588 and ILL 2601. For each SNP, the first variant given in the table correspond to the allele present in ILL 5588 and the second to that from ILL 2601.

Position	SNP	Position	SNP	Position	SNP	Position	SNP	Position	SNP	Position	SNP	Position	SNP	Position	SNP
295	G/T	7040	A/T	19919	T/C	22681	C/T	25765	A/T	28500	T/G	32822	C/T	35816	T/C
782	A/G	8084	A/G	19938	C/T	22734	C/T	25781	T/C	29655	T/C	32846	C/T	36016	A/G
990	C/A	8085	T/A	20002	C/A	22878	C/T	26049	A/G	29982	T/A	33235	A/T	36347	A/G
1362	G/A	8086	T/A	20191	C/T	23592	T/C	26258	A/C	30294	G/A	33405	T/C	36374	G/C
1454	G/T	17191	C/A	20249	T/A	23633	C/T	26682	C/T	30331	C/T	33434	C/T	36637	T/C
1904	G/T	17605	C/T	20254	T/C	24343	C/T	26719	T/C	30362	C/T	33449	A/G	37102	A/G
2163	A/G	17801	A/T	20265	C/T	24519	A/T	26835	A/T	30466	C/T	33545	C/T	37280	G/A
2444	G/A	17874	C/T	20467	G/C	24599	A/G	26836	G/T	30598	T/C	33730	C/T	37493	C/T
2643	A/G	17897	C/T	20489	C/T	24690	A/C	26837	A/G	30619	T/C	33747	A/G	37531	C/T
2774	A/G	18161	A/G	20594	C/A	24726	C/T	27103	A/C	30689	C/G	33836	A/C	38597	A/G
2834	A/T	18564	T/C	20804	A/G	24955	A/G	27409	C/G	30723	T/C	33968	T/C		
2882	T/A	18625	C/T	21328	A/T	25245	C/A	27573	A/T	30827	C/T	34279	T/A		
2902	G/A	18783	A/G	21431	C/G	25304	A/T	27623	C/T	31237	T/C	34329	T/C		
3728	G/T	19068	A/G	22069	G/A	25338	A/G	27827	A/G	31533	A/G	34377	C/T		
5015	G/C	19100	C/T	22240	A/C	25400	T/C	27897	A/C	31866	A/G	35310	G/A		
5022	A/G	19141	G/T	22405	G/C	25517	T/A	28121	T/A	32104	T/C	35396	C/T		
5258	T/C	19524	A/T	22478	C/T	25524	C/T	28126	A/T	32510	C/T	35712	C/T		
6214	T/C	19554	C/T	22539	T/C	25559	C/T	28161	C/A	32785	T/G	35714	A/G		

Supplementary Table S9. Description of all indels found in the 38926 bp-length alignment between the *FTa1-FTa2* sequences from lentil accessions ILL 5588 and ILL 2601. For each indel, the first variant given in the table corresponds to the allele present in ILL 5588 and the second to that from ILL 2601.

Indel	Indel start	Indel end	Length	Indel	Indel start	Indel end	Length
TT/-	141	142	2	-/Var3 [a]	27410	27515	106
-/A	520	520	1	G/-	27759	27759	1
-/Var1[a]	535	779	245	-/Var4 [a]	29263	29548	286
-/GGGGTGGGGGGG	2779	2790	12	GCGTC/-	29755	29759	5
-/TTC	3680	3682	3	TGTTTTAAATATAAAAACTT/-	29878	29897	20
T/-	7012	7012	1	Var5 [a] /-	30386	30437	52
-/T	7262	7262	1	-/TCT	31210	31212	3
AATAATATAGTTATTTTTTA/-	7734	7753	20	-/TTC	32986	32988	3
ATAT/-	8053	8056	4	-/AG	35740	35741	2
Var2 [a] /-	8243	15683	7441	TTT/-	36817	36819	3
A/-	17716	17716	1	-/T	37009	37009	1
-/A	18680	18680	1	AACAGT/-	38440	38445	6
-/TT	26458	26459	2				

[a] Due to their excessive length, the sequences corresponding to these variants are shown separately in the next page(s) of this document

Variant 1 sequence

TCTCTTCAAACATTTACAAACAAACACTATTTTCAAGGTAAAAACACGGATTTTCAAAAGGTTCCAGTGGAGTACCACATATGTGAGGGGTGCTAATACCTTCTCTTCGCATAAC
CAACCTCTAAACCCTTATTTCTTTATCTTGTGGGTTTTATTTGCTCTTTTCCCATTTTCCTTTTAGAAACAATAAAAGTAAGGTGGTGACTCTTGCTTTATGAGTTAAGTTAATTAA
TAGCTTAAACTCA

Variant 2 sequence

TTCAAACAGCAGTTGTAACATATAGTTAACTGAAGAAGCTAGGCTTCAAAGAGATGATTAAAAATTTAAGAACTAAAGAAAAATAAATAAATAAAAAACGCTAAGAAATGATA
TACAAGTGGAATCCGCAATTAAGGTTTCTATTAAAAAAAATAGAAGAAAATCATAAATAGTAGTATTAATATTTTAAAATAATGTGATGGTTAAAAAGGAAATCTATATGCTAT
TGAATAGTCATTTTCTACTAATTATTTGATGATTGTATAAACATTACATCTTATATCTATCGAGAGTAGAGCTCTGGTCTACTTTCTCAAACCATTAATCATACTACTTGATCAATG
TAGGGCTATTCAGGACCTTTTCCAAAAAATAAATAATAAACAAACAACCGCTCGCTAGTTGCTATTTATGTAGTTTAACTAAGTGCAAGAAAGCAGGAACGTGTGAAAGACCA
CATCTCTCACGCTTGTAGTTTTTTCTTCTCTCAAAAATATTTATTATTTAAAATTATAATATATTATATGTTAGAAACAAAACCCTTTGAACTAGATAATATCTTCAACTGGAGGAG
AAAAAAGAAAATATCTCCATCTCACTGTTCTGCACACAGTGGCTACTACTGTCATCTATGAAATCACCTTTCCTTGTTCGCAAGGAAATTACAGTTTCTACACAAATATTTCATCT
CATTCTAAAATCTTACTTTTCCCCCAAAACATAAACCACAGAAACAACGTGAAGATGGAAGAATGTAAATGTGAAGTGAAAAAGTTTTTCACTTAAATCAGGAACCCTAGCAAA
CCACAGTTCACTGAAAGAGACGACAGTGGCAAATTTACCTGATACCACGGCAGTTATTGTGGATGACCTCGTCCTGTTACAGTGAAAATCTGATCATGTGGCGGTTCTGTTGA
CAAATCTGTAACTGTAGTTCTGGGAAGATTTTCACCCTAGGTTTTCCTGGATCTATAGTTCTACAACGTGTTAGATTTATGACGAGGATGAGTGACTTCTGGTTTTAATTTTAGA
TATATATTTATCTGAGATCTAGTTTTGTTGTAAGGGGTGTGCTTTTGAAATCATGAAAATTTGCAGGTAAATTTAACACTGTTTGTGATTTAATAGAAAATAACTTTAACTTTTTT
TTAAAATATTGAATGATCTTTGGATTTAATCATTTCCATTTTATTCATCTGTTTTATTTATTTATTTATTTAATTACGACGTGCTTTGCTTTATAGTTTTTTTCTTTATAGGGTTAAAT
TTGGCATTAAATTGTGGTTATCCAATTCCAGATCGATCTACTGGAAGGTATTTTACTTCCTAAAATTTCTATAGTTAAGTTTACACTCCTAATGTTGGCTTGGTGATTTAGTTTTG
TCCTATAGTTTAGTCTTTTTACACTGAATCTATAATGATTTAATTCTAATTATAACTACTTTTATAATTGTATAAATAAGAATAATTGTGATTTATTTGTAGTATAGAAATTTTTGCA
GTGCTTAACACACGCTAAAGCATGATCATATATATATGTTAATTTGATTAATCAGTTAATTGTATTTCTGCTAGCTGTCAAATATTTTTATTACTACTATATCATTTTAAGTTTCAATT
TAATGGTTGTATGAATAAAATCTAATGTTCATTAATATATTATTATTATTATTATTATTATTATTATTATTATTATTATTATTATTATTATTATTATTATTATTATTATTATTATTATTATAAAGTTTAGAAGT
TATTTACATTTTATCCAAAAAAGAAGGAAGCTTACTTACATCAATTGAAATAAGAGTTGTCTATGCTCAAACTCAAATCATTAGTCATTCTCATTCAGGTCCAATTGTTAGGGAT
AGTCTGGTACTTTAACAATGAAAAAAAGAAATAAGTGCTGCCCGCTGCCCGCTGGTGCTTCTATATTAATATTAACCAATTGCAGAAACAAAGAAAAGCCGGAACATGTGCAA
TACCATATCTTCATCCCATGCTTTAGAGTTTTGTAAATTTCAAACAAATTAAAATATCTAAAGTAACTATAAAAAAAATAAAATATCTAAAGTAACTATAAAAAAAATAAAATATCT
AAAAACCCTTTGAACGAGTTAATATCAAGGAAAAACTAAAATATCTTCAGTGCACTGTTTTCACCAAAAAAATAAAAACATAAAAAACTCCAGTCCACTAGTGTTTTGCACTCA
GTGGCTATGATGACCTATGTAATCATCTATACTAGATAGTAAATAACAAGTTCTACACAAATTGTTCATTTTTTTCTAAAATTTCTTCTAATCTATAGAAGAAAGGTGAAGAATG
TAAAATTTAATATATGTGAAGAATGTGAAAGAGTTCTCACATATATAAAGAAAAAACTCTAACTAACTAAATAACCAATATTTCACTGCAATAGATGGTGGTGGCAGAGCGGA
TATACCTTATGCTTCGACTGCGGTGGTGGAGGACTTGGATAATCAGATTATGGTGGTGTTGTTGTTGAAGGTTTAGCTGCAGTTCCGGAAGGATTTTCACTTCATGATGCTCTT
GACGAGTATGAGTAATTTAATATTGTTGTAGAAGTTGTGGTGAGTTCAACAAGCATTAAGTATACTTTTCCAAATATTTTGGTGAGTTGAATTTTAGATATTTAAAATATAAGTC
CATTTTTATTTATATATCTTATACTATTTGCTTTCTATTTATGCTTGTCTTTTGGAAGTGGAATTGGGGCAACCTACACGTGTCTTACTTTATAGGGTTGATAATGGCATTGAATTG

CTCTGATCCATGTCGATCCAATCGCAGGTATTTTTACTGGCTAAAACTTTTATAGTTTATAGTTAATACTTAATACTCAATGTGATGTCGGCTTGGAGAAGTTTTGTCCTATTCTT
GGAGTAGTGTGACTAGTGTAACTATATACTCTTTCCCAAAATAGATGTTGAAAATAAAAATCACATTTAATAAAATAAAAGTACACAATAATTGAATTATGGTTTTTTGTATTTT
GTTAAAAAAAAATTAAAAAGATTATTGTTAATTATTTATTGACTATGGTGAAAATGAATGAGATTTTATATTAATAATCAACAATTTTTTTAAAAAAAGGTCTTGTATATTTATGT
TATGTTTGGATACACATTGAAAATCATAAAATGCGTGTCTAACATACACTGGTCTTTAAAATGTGTATCCAAAATATGCACTTAGACCATCTCCAATGGGAGTTCTTTGCAAGTT
CTTTAAAGAACCAATTATATATTTTCAATATTTTAATCAATGGAGTGTGCCAAGTTGGAGAACCATTACTTGCTGAAGCAACTTTGGTGATGTGGTTCTCAATAAAATATGATGT
GTCTTAATGGATTTCACAAAAAACTCAAAAATGAGTTGCACCATTGGAGATGCTCTTAGTTTGATGAAGATATATGTTGTTCTTTTTTAATAAATTTGGATAAGAAATATGTGTT
TTTCTTGTTTGTTTATATTTTAGGGCGGAGGTACAAGGTAGTATTATTGGGTCAAAATGTATTTTGGTTCGCGAAGGAAAAAAAATTGTATGCTTCATTATCTATTGTAAGTTTAT
GTTTAGTTTCACGTTTAAAATTGATTACGGATGGTTAGAATTGATTTTGACATTTTTATTTGTCTTTGAGTAAAATTGATTTTGATATGTTTGATTGCTATAGAGTTGGATTAATT
ATACTTTTCATAATTAATTGTTCTTGAAGCTAAATTTTTTAACTTTTACGTGTAAACAAGATTTTTATTAAGGGTGACAAAACATATCTCGTTCGCTGGACATGTTTGTTTTGCCCG
CACTATTTCGCAGGATATTCCAAGATTTTAGATCAACACTCTCTAATATGTTGGATTTGCTTCCACTTCGCCATAATTTTTTGTAGGACGAGTCAAAGTTTTAGGCTTGTGCCCTC
AACTATGTCTCTCTCGTCTCACCTTTTTTTTGTGCAGGTTTTTGTGTGGCGAGTCTAAACCGAGCGAGTATGTCCGACACTTAAGCTTAATGTTAACCTCACTTCTACAAAAATAT
ATGCAAACATAAATCAGTTTATCTTTAACTCATTTTTAGAAAGAATCAATTTTATGTGAACTATTCACTCTATCAACTTTCTTTTATGCAAAACCAAACACACATTAAGCATTAAGT
TATATTGATATGTTACTTTATACTTTCATTCATATGCAAATTTAACGTGAAAAGTAAGAAGCTTTAATTTGGAGCATTGGCCAAATGCACAACGGATGGATATCCCATAGTAAAA
TAGAGAAAAAAGATCTTATTATCGGTAGAAAATAGATGCCGAGGGATTTAGAATTCTTAGTTGAATTTGATTTAAAGCTAAACTTCTAAGGTCGTCTCCGACCCACATCTTGAG
ACAGGGTTGCATACTTCGATTTTGTAAGTCTACTTTTGTCATATAATGATAAGATATTTGATGATAATTACTCTAAATGTAGTTCTTTATTCATCTTAATTAACAAATATTTCACAA
TAATGTCTTGTGTTAGAATGTATGATGAGTTTATTTGTCCACTTCTATGAAATCCAATTGATTTTTTAGCAAGTCCTTACTATTTTTAAATGATAAGGAATGCATTTCCTACATATC
GTTGGTTTAAGATGTACAAGAATATTAAAGCACTAAGAGTTTCACCTAAATCTACTTCAATGGTTAAAAATTGAAACTAAGTCCTCGTAGATAAGAGATGAAAAAAAAACATGT
GTTAAAGATACTAATGTACATATAGTTAAAATTCAGTCTCTCTCAATATCACAAACAAAAATTCTATTTGAGGGTGACAAGCTGACATTTCTGCTTTCTCAAAATCTAGGAGTCC
AACCCATTGTGTTCCCTCTAAAACCTATGAGTGACAAACATCTATGTCTACTATATTTAGGCACGAGTTTGTTAAATCCTTTTAAAATGGTCTCATGGACAAAGTAAAGGGGATA
TATTTGATACCTTAGTCTCGTTATGTTAAAAATAGAGATAAATGAGTAGATCAATCTATTTTGTCACTATTTCTTCTTATCTCTTTTTTAAGCATGCATATTAAGAATAGGTAAAA
ATCCTTCTTGATTGTTTTAAATTGAGGTGATTGTCGAAGGTATTTCTTGTTGTAAAAGGGTTCGAGTACCCCTTGTACTCGTTTGAATGTTTATTTAAAAAAAAATATAGGTAAA
GGGAAGTTAAGAAGGGAAAAAAGACATCAAACAAAAATTAAAATCAATGTTTCAGATGATGTAGTCAATTGCTGCCATTTGGGCTAATCAATGAACGTGTCATGAAAATCTTG
GGTTTTGATATATAATGTTGGCTCACTCTAAAATACTTTGTCGATGAAATATTAAATGAATTCAACTGTATCATGCTCGAAAAACTTGTTTTAGACCATATGATTATTGTAGTTTG
AAAACTATGCATTTGTTGCCTATGTCGTTGCTCCCTGCAAATCTCTTATTCGATACTTTATTTAGAAAAGCTATTAATCTGGGCTTATGTTTTTGTATTGACATCATGCTTTAGATT
GTATAAACCAATTTAATGCCAATGTTTCTTACTTCACTTTGCTTAAAAAAAAAAATAAATATGAAATAATTATTACAAAAATCACGATTTCTCATTAAAATTAATAAGATTTTTTTC
TTTGAGAATTATGATTGAAAATTGAACTGTCAACCATATTAAATATTAAACTTTCGTTTATCAACTTATTAATAAAGAAAGATCTAAGTGATACCCTTATCCTAATAATGAAAATT
CACCTTAGATATATCTAAATACTAGATTTTCCAGCTCTCACAAAAGCTTTTGGCATATATATATATATATATATATATATATATATATATATATATATATATATATATATATAATATGA
CAAAAATATATTAAACTTAGTAATATGACACCGCCAATAAGTCTTCATCAATTATTTTTATGACAAAATCAATTATTAGATTAAAATCTATTATCATATAAATCATGTCTATAAAA
CTTAATACCAATAAAAATTTATATGATAAATAAAGGGAAAGATCAAATTAACGTTAATAATTATGCATTTTGTTGACGTGTCGAATAATATTTTATTAATGTTCAATTTTATAGAT
ATAATTTATACGATAAAAATTTTAAATTAAATCGTAAATTTTTTTATATGAATAAACGTTAAATCGTAAATTTTTTATTGATGTACGATATTATATATATATATATATATATATATA
TATATATATATATATATATATATATATATATATATATATTCTAGTAATATATCTCTTGACTAAATTATTTTTATTATGATTATAACAGAAATATAAAAGACCACGACCCACCCGACCCA
TACAGAGTTTTCATAGGTTTTTTTTTGACAAGGTCTTTTTTGTTCTTTTGTGTTTGCATTATTTAAATTTTCAAGTATACTTTCACCATATACAGTCTGGACAAAGAGTTTAACATC
CAATAAACACTTGAATTTTATGTATTTTCATCTAAATACAGTAAATTATCATAATTGTCAAATAAAAAAAACATAATAATAATAATAAATGCGAAACCCTGAACTGAAAAATATC

TAGTTCTGAAAGGCGGTTTTCTTGTGCATAGTAGAAGCCGTCACTTACGAAATCACCCCTGCAAGCCACGCACGCTCACCCCACCTCACAACTTGTGCACGATTTTTCCCTTTAA
CCATTAGCCGCTCTATTTTTCATCTCTATCCCAATTTTTCTCATATTCTCCTAAATCAGTAACAGAATCACAAAACCAACTTAAACAAGGACACAGTGAGTTGAGAGTGTTCACAC
TCGAGTCAAAACCCTAACTTACCAGCGATCCGCCATAACTGACGCCGGCGGTGGTTTGACGGTTGTATCCGCGATGGTTGCCGAAAAATCCGTCGTGTGGAAGTGACAATCCG
TTTTGACGGTAGTGATCTCAACGGAACAGAAGGTTGCAGTTCCGGAACAATTTCAACCGAAGGATGTACTTGATCTACACACTTTGCTGGTTTTGATAGTGAGTATGTGTAATT
TCTGGATTTGAGATCCACTACGTGAAGTTCACGCTGTTTGAGTTTTTAAATATTTTGTAACACTGTTTTGTTTTCTTCTTGCAAGTTTTTTGATCCTCTGAAATCCTTGAACCTTCG
TTGGTGAAACTTCATGCTCATTTTTTCTAAACGAAAAAATATATATCTAATATAGCTGTAAGTTTTTTTTTGTTTTCAAAACCGTATTGTATATGCTTTGAATAGGCAATATTTACC
GATGATTTATTTTGAATTGTCCTAAATTAGTTTTAAAGTTATTCTTGGAGTGCAGGGATTATATCATGAGTTATACACTTACACTGTCATCATTCCAGTGTAGGTAAGTTCATGCC
CTTTTATTCTTCTTTTATTTAAACTTTCCTTTTAAGTTTTATA/-

Variant 3 sequence

TTCTTCTTCTTCTTCTTCTTCTTCTTCTTCTTCTTCTTCTTCTTCTTCTTCTTCTTCTTCTTCTTCTTCTTCTTCTTCTTCTTCTTCTTCTTCTTCTCCTCTAGCCTTTTCA

Variant 4 sequence

GATTTATGATGTGTTATTAATTTTAATGCTTTATTGTTTTCTGAAAGGCAAATAAGATATGTAATAGATAAAAAGATAAAAACTATTTTACAAACAATCAATAGAAAAATTCAAG
GAATACATAAGAAGTATTAGATCCGAGATCAAAAACGAACATAAAAAACGCAAAACATCTACCAATTACAATAAACTCAGCCGACAATGAACTAGGGCTACCCAATGTGAGG
GAGAACAAAGAAAACACACATAAAAACTTCTTTCTGCTTCAAACGAGACTAGGCTTCTC

Variant 5 sequence

AATGTTTAATATTTCCAACAAAATTCCCCTGTAAACATTAAATGCTTCTTCT

Supplementary Table S10. Mean and standard deviation values obtained for days from emergence to first flower (DTF) and node of the first open flower (NFD) in lentil lines ILL 2601 and ILL 5588 grown in 4 different conditions.

			DTF		NFD	
**Accession**	**Treatment**	**N**	**Mean**	**Std. Dev**	**Mean**	**Std. Dev**
**ILL 2601**	**NV-LD**	12	35.5	2.5	8.2	0.9
	**V-LD**	19	37.9	3.3	8.0	1.0
	**NV-SD**	9	65.7	5.7	10.8	0.8
	**V-SD**	10	62.8	8.6	10.6	1.3
**ILL 5588**	**NV-LD**	13	41.4	2.8	13.6	1.2
	**V-LD**	21	39.1	2.5	12	1.5
	**NV-SD**	15	-[a]	-[a]	-[a]	-[a]
	**V-SD**	23	71.8	4.8	13	1.4

N= Number of plants; LD = Long days; SD = Short days; V = Vernalized plants; NV= Non-vernalized plants

-[a] Plants were unable to flower/set pods under this conditions during the scoring period.

Supplementary Table S11. Primers used in the PCR-based marker of the 7441-bp deletion in the *FTa1-FTa2* intergenic region.

Primer name	Sequence
F1	TGGGCTTGATACTTTGTACTCC
F2	TCTACACACTTTGCTGGTTTTG
R	CCATCACAATTCAAAGCAATG

PCR conditions: PCR was performed in a final volume of 40 µL containing 250 ng template DNA, 10 µL of 5x reaction buffer, 10 mM dNTPs, 0.2 µM of each primer, 50 mM MgCl2, 0.2 µL of MangoTaqTM DNA polymerase (Bioline, Australia) and autoclaved Milli-Q water to final volume. Reactions were performed in a thermal using the following program: an initial denaturation of 5 min at 94°C, followed by 40 cycles (94°C for 45 seconds, 58°C for 30 seconds, extension of 45 seconds) and a final extension of 10 minutes at 72oC.

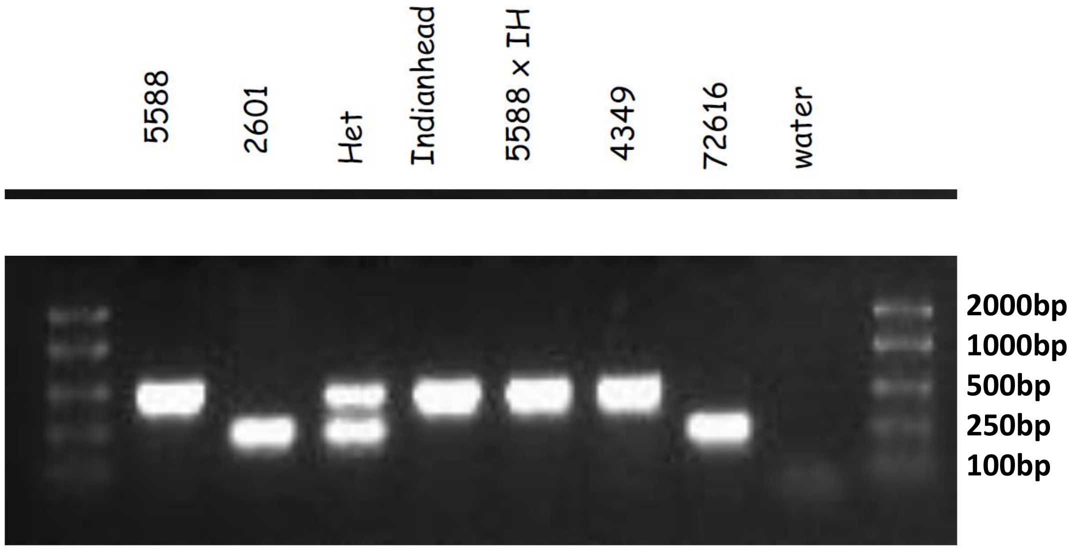

Supplementary Figure S9. Visualization, in a 2% agarose gel, of the PCR products obtained with primers described in supplementary table S11 tested in different lentil lines. The ~500 bp and ~250 bp bands correspond to primer combination F1/R and F2/R, respectively.

Accession	species	Country	Latitude	Longitude	Altitude	Accession	species	Country	Latitude	Longitude	Altitude
ILL 1823	L. culinaris subsp. culinaris	Afghanistan	36.9	70.9	1525	PI 345631	L. culinaris subsp. culinaris	Russia	53.2	45	220
ILL 2153	L. culinaris subsp. culinaris	Iraq	36.8	43	460	PI 345552	L. culinaris subsp. culinaris	Russia	42	47	3200
ILL 2214	L. culinaris subsp. culinaris	Lebanon	33.8	36	910	PI 368649	L. culinaris subsp. culinaris	Serbia	45.4	19.2	620
ILL 223	L. culinaris subsp. culinaris	Iran	38.7	46.3	1360	PI 374116	L. culinaris subsp. culinaris	Morocco	31.8	-	480
ILL 2276	L. culinaris subsp. culinaris	Lebanon	33.5	35.4	30	PI 420924	L. culinaris subsp. culinaris	Jordan	32.1	35.8	860
ILL 2601	L. culinaris subsp. culinaris	India	23	-	-	PI 426775	L. culinaris subsp. culinaris	Pakistan	27.6	68.1	42
ILL 4349	L. culinaris subsp. culinaris	Canada	-	-	-	PI 426788	L. culinaris subsp. culinaris	Pakistan	31.9	73.1	180
ILL 4370	L. culinaris subsp. culinaris	Iraq	33.3	44.4	40	PI 426797	L. culinaris subsp. culinaris	Pakistan	32.3	74.4	234
ILL 4605	L. culinaris subsp. culinaris	Argentina	-	-	-	PI 472291	L. culinaris subsp. culinaris	India	26.7	85.2	50
ILL 5065	L. culinaris subsp. culinaris	Jordan	32.6	35.7	300	PI 472311	L. culinaris subsp. culinaris	India	22.4	88.1	10
ILL 5588	L. culinaris subsp. culinaris	Jordan	32.1	35.7	700	PI 472317	L. culinaris subsp. culinaris	India	14	77	600
ILL 5895	L. culinaris subsp. culinaris	Ethiopia	7.1	39.1	2440	PI 472328	L. culinaris subsp. culinaris	India	27.6	81.6	126
ILL 5976	L. culinaris subsp. culinaris	Cyprus	34.8	33	260	PI 472343	L. culinaris subsp. culinaris	India	26.3	78.2	180
ILL 6005	L. culinaris subsp. culinaris	Argentina	-	-	-	PI 472359	L. culinaris subsp. culinaris	India	26	93	60
Indianhead	L. culinaris subsp. culinaris	Canada	-	-		PI 472578	L. culinaris subsp. culinaris	Iran	35.8	51	1300
PI 297774	L. culinaris subsp. culinaris	Greece	40.9	23.7	140	PI 472625	L. culinaris subsp. culinaris	Iran	35.2	59.4	1340
PI 297779	L. culinaris subsp. culinaris	Greece	39.7	20.8	580	PI 509333	L. culinaris subsp. culinaris	Turkey	37.2	38.8	650
PI 297789	L. culinaris subsp. culinaris	Greece	38.3	20.5	700	PI 509409	L. culinaris subsp. culinaris	Turkey	39.7	35.8	1300
PI 298122	L. culinaris subsp. culinaris	France	48.3	-	60	PI 513328	L. culinaris subsp. culinaris	Pakistan	25.3	68.8	20
PI 300248	L. culinaris subsp. culinaris	Syria	36	37.2	400	PI 533693	L. culinaris subsp. culinaris	Spain	42.3	-	860
PI 300250	L. culinaris subsp. culinaris	Syria	36.4	37.2	460	PI 606600	L. culinaris subsp. culinaris	Nepal	29.6	81.3	1368
PI 339281	L. culinaris subsp. culinaris	Turkey	38.2	29	780	PI 606610	L. culinaris subsp. culinaris	Tajikistan	40.4	69.9	560
PI 339293	L. culinaris subsp. culinaris	Turkey	38.3	33.6	920	PI 606615	L. culinaris subsp. culinaris	Russia	45.8	38.6	-
PI 357227	L. culinaris subsp. culinaris	Macedonia	42	22.2	450	ILWL 7	L. culinaris subsp. orientalis	Turkey	38	-	-

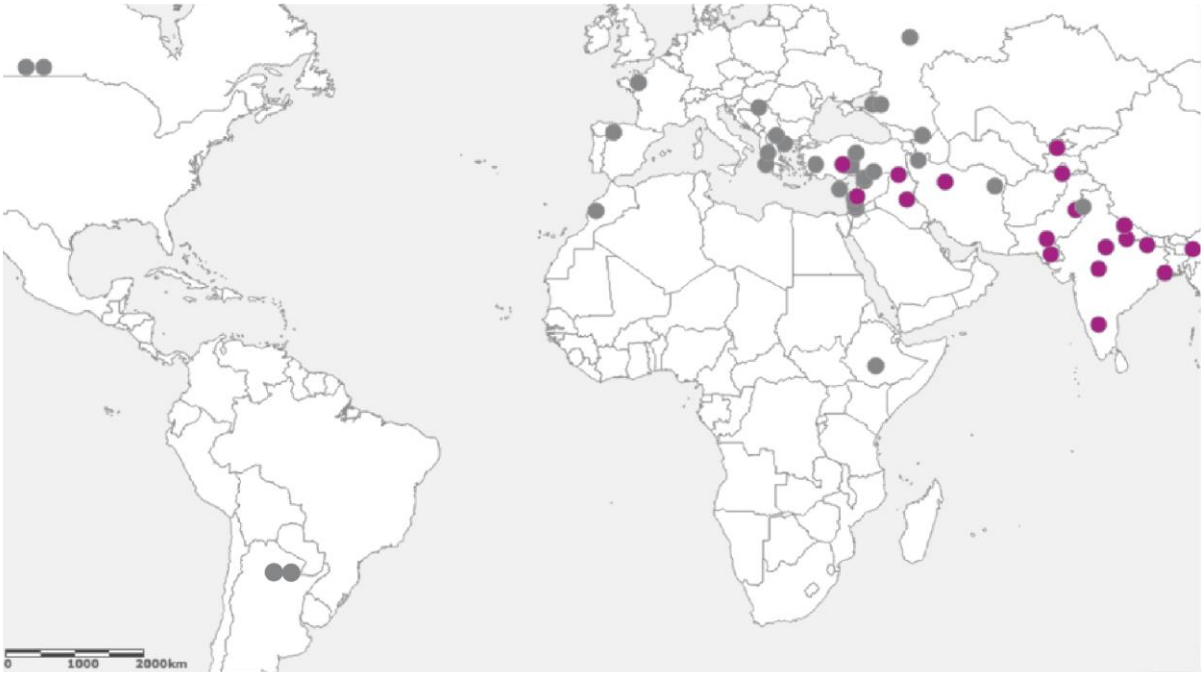

Supplementary Figure S10. Incidence of the 7441-bp deletion in 48 lentil lines (University of Tasmania lentil collection) with diverse geographical origin. Purple or grey circles in the map represent accessions carrying (ILL 2601 allele) or not carrying (ILL 5588 allele) the deletion, respectively.

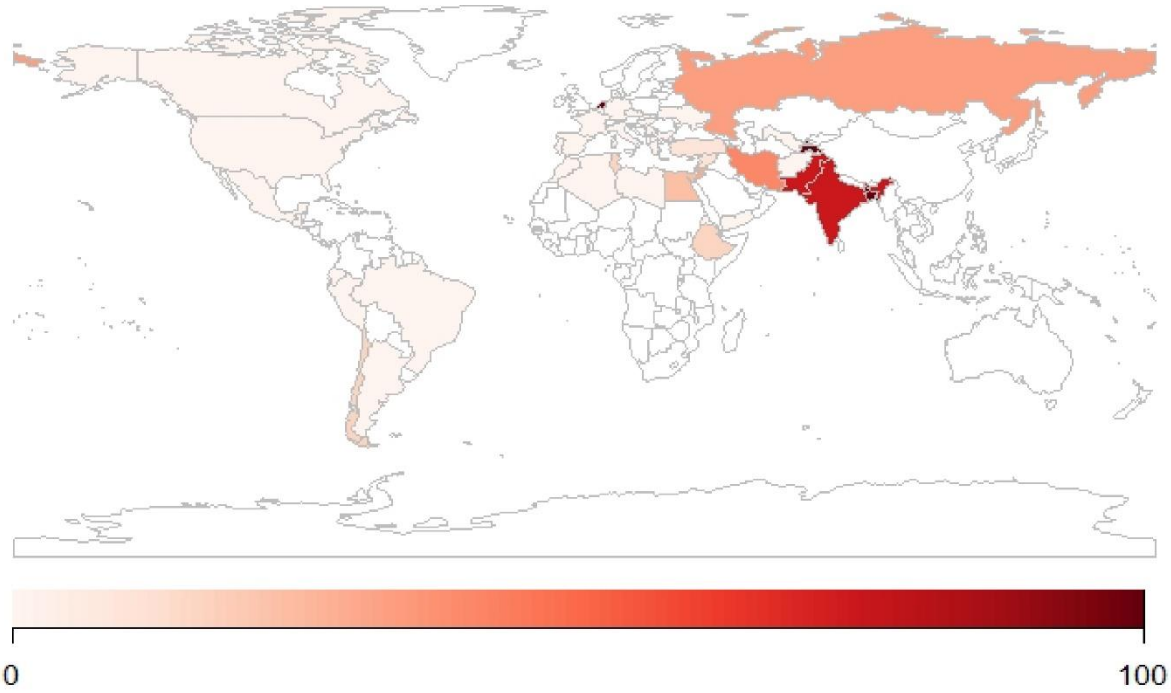

World				
	N	WT	DEL	%DEL
World	324	249	75	23.1

Central Asia				
Country	N	WT	DEL	%DEL
Tajikistan	2	0	2	100.0
Afghanistan	7	7	0	0
Uzbekistan	1	1	0	0

South Asia				
Country	N	WT	DEL	%DEL
India	28	7	21	75.0
Pakistan	8	2	6	75.0
Bangladesh	4	0	4	100.0

Africa				
Country	N	WT	DEL	%DEL
Eritrea	1	1	0	0
Ethiopia	24	20	4	16.7

America				
Country	N	WT	DEL	%DEL
USA	6	6	0	0
Peru	1	1	0	0
Brazil	6	6	0	0
Argentina	3	3	0	0
Canada	28	28	0	0
Chile	12	10	2	16.7
Guatemala	2	2	0	0
Ecuador	1	1	0	0
Mexico	6	6	0	0

Middle east				
Country	N	WT	DEL	%DEL
Jordan	10	7	3	30.0
Yemen	2	2	0	0
Syria	11	10	1	9.1
Iran	49	29	20	40.8

Mediterranean				
Country	N	WT	DEL	%DEL
Greece	5	5	0	0
Egypt	13	10	3	23.1
Morocco	5	5	0	0
Spain	4	4	0	0
Tunisia	6	5	1	16.7
Algeria	2	2	0	0
Libya	4	4	0	0
Portugal	2	2	0	0
Turkey	20	18	2	10.0
Lebanon	3	3	0	0
Israel	2	2	0	0
Italy	3	3	0	0
Bulgaria	1	1	0	0
Macedonia	4	4	0	0
Serbia	1	1	0	0

Europe				
Country	N	WT	DEL	%DEL
Russia	3	2	1	33.3
Ukraine	1	1	0	0
Belgium	1	1	0	0
Netherlands	1	0	1	100.0
Czech Republic	4	4	0	0
Hungary	3	3	0	0
France	4	4	0	0
Germany	2	2	0	0

Other				
Country	N	WT	DEL	%DEL
Unknown	2	2	0	0
ICARDA	11	7	4	36.4
USDA	5	5	0	0

Supplementary Figure S11. Incidence of the 7441-bp deletion in a panel of 324 lentil lines with diverse geographical origin. Colour in the map represent percentage of accessions that present the deletion. Countries without colour were not represented in the panel. N = total number of accessions; WT = number of accessions with a wild type allele (no deletion present); DEL = number of accessions with the 2601 allele (deletion present); %DEL = percentage of accessions carrying the deletion.
